# Tirofiban on First-Pass Recanalization in Acute Stroke Endovascular Thrombectomy: The OPTIMISTIC Randomized Clinical Trial

**DOI:** 10.1001/jamanetworkopen.2025.5308

**Published:** 2025-04-17

**Authors:** Longting Lin, Feifeng Liu, Tingyu Yi, Yueqi Zhu, Jianhong Yang, Yanxin Zhao, Feng Wang, Guangyu Xiang, Chen Chen, Yaping Xiao, Hao Shen, Luran Xu, Yuming Long, Yue Zhang, Zhengyu Huang, Chushuang Chen, Leonid Churilov, Mark W. Parsons, Wenhuo Chen, Gang Li

**Affiliations:** 1 Department of Neurology, Shanghai East Hospital, Tongji University School of Medicine, Tongji University, Shanghai, China; 2 Sino-Australian Neurological Clinical Research Cooperation Centre, Shanghai East Hospital, School of Medicine, Tongji University, Shanghai, China; 3 South Western Sydney Clinical Campuses, University of New South Wales and Ingham Institute for Applied Medical Research, Sydney, New South Wales Australia; 4 Department of Neurointervention, Zhangzhou Affiliated Hospital of Fujian Medical University, Zhangzhou Municipal Hospital, Fujian, China; 5 Department of Radiology, Shanghai Sixth People’s Hospital, Shanghai Jiao Tong University School of Medicine, Shanghai, China; 6 Department of Neurology, The First Affiliated Hospital of Ningbo University, Zhejiang, China; 7 Department of Neurology, Central Hospital, Shandong First Medical University, Shandong, China; 8 Department of Neurology, Seventh People’s Hospital of Shanghai University of Traditional Chinese Medicine, Shanghai, China; 9 Comprehensive Stroke Centre, Xuchang Central Hospital, Henan, China; 10 Department of Medicine, University of Melbourne, Melbourne, Victoria, Australia; 11 Department of Neurology, Liverpool Hospital, Liverpool, New South Wales, Australia; 12 Department of Neurointervention, Zhangzhou Affiliated Hospital of Fujian Medical University, Fujian Medical University Union Hospital, Fujian, China

## Abstract

**Importance:**

Tirofiban is a nonpeptide antagonist of the glycoprotein IIb/IIIa receptor that inhibits platelet aggregation selectively. Despite widespread use in acute ischemic stroke in conjunction with endovascular treatment, the role of tirofiban is uncertain.

**Objective:**

To assess whether intravenous tirofiban initiated before endovascular thrombectomy improves thrombectomy outcomes by achieving first-pass recanalization without intracranial hemorrhagic risk.

**Design, Setting, and Participants:**

This was a multicenter, prospective, open-label, blinded, end point phase 2 randomized clinical trial that enrolled patients from 7 Chinese hospital stroke centers between April 30, 2021, and July 16, 2023. Eligible patients were aged 18 to 85 years with large vessel occlusion (internal carotid artery or M1 or M2 segment of middle cerebral artery) and were considered for endovascular thrombectomy within 24 hours of ischemic stroke onset or last known well time. Patients with a history of atrial fibrillation or treated with intravenous thrombolysis were excluded.

**Intervention:**

Patients were randomly assigned (1:1) to intravenous tirofiban (10 μg/kg bolus plus 0.1 μg/kg per minute infusion for 24 hours) or standard care before endovascular thrombectomy using a web-based, computer-generated randomization procedure.

**Main Outcomes and Measures:**

The primary outcome was the proportion of patients achieving first-pass recanalization without symptomatic intracranial hemorrhage. First-pass recanalization was assessed by reviewers blinded to treatment allocation and predefined as successful recanalization of a targeted vessel after a single pass of thrombectomy. The primary outcome was analyzed by modified Poisson regression with robust error estimation, adjusting for time from onset or last known well time to randomization and vessel occlusion site.

**Results:**

Among 200 patients randomized (median age, 66 years [IQR, 58-72 years]; 146 male [73%]), 102 were allocated to receive intravenous tirofiban, and 98 were allocated to receive standard care before endovascular thrombectomy. In the intention-to-treat analysis, 64 of 99 patients receiving tirofiban (65%) and 46 of 95 control patients (48%) achieved the primary outcome of first-pass recanalization without symptomatic intracranial hemorrhage (adjusted risk ratio, 1.34 [95% CI, 1.04-1.73]; *P* = .03). Incidence of symptomatic intracranial hemorrhage was 0% (0 of 101 patients) in the tirofiban group vs 6% (6 of 98 patients) in the control group (unadjusted risk difference, −0.06 [95% CI, −0.11 to −0.01]).

**Conclusions and Relevance:**

In this randomized clinical trial of patients who had acute ischemic stroke with no history of atrial fibrillation and no prior intravenous thrombolysis, intravenous tirofiban administered before endovascular thrombectomy increased the likelihood of first-pass recanalization without symptomatic intracranial hemorrhage. These findings suggest that neurointerventionalists may consider this pre-procedure antiplatelet treatment to facilitate endovascular thrombectomy.

**Trial Registration:**

ClinicalTrials.gov Identifier: NCT04851457

## Key Points

**Question:**

Does intravenous tirofiban initiated before thrombectomy increase the likelihood of first-pass recanalization without increasing the risk of symptomatic intracranial hemorrhage in patients who have had an acute ischemic stroke?

**Findings:**

In this randomized clinical trial that included 200 patients with anterior large vessel occlusion who have had an acute ischemic stroke, with no history of atrial fibrillation and no prior intravenous thrombolysis, the proportion of patients achieving first-pass recanalization without intracranial hemorrhagic risk was 65% with tirofiban compared with 48% without tirofiban, demonstrating a significant difference.

**Meaning:**

In this study, intravenous tirofiban administered before endovascular treatment in patients with anterior large vessel occlusion who had an acute ischemic stroke increased the likelihood of rapid recanalization without symptomatic intracranial hemorrhage, particularly among patients with intracranial atherosclerotic disease.

## Teaser

This randomized clinical trial assesses whether the use of intravenous tirofiban before endovascular treatment in patients in China with anterior large vessel occlusion who have had an acute ischemic stroke increases the likelihood of first-pass recanalization without intracranial hemorrhage.

## Introduction

For acute ischemic stroke caused by large vessel occlusion, endovascular thrombectomy has become a mainstay of acute treatment.^1^ Despite a high recanalization rate with improved thrombectomy devices over the past decade, endovascular thrombectomy fails to yield a good clinical outcome in half of patients who have had a stroke.^2,3,4^ Delayed recanalization with endovascular thrombectomy is associated with a less favorable clinical outcome.^5^

First-pass recanalization has been proposed as a new outcome measure for thrombectomy success.^6^ It has been included as an angiographic efficacy outcome in thrombectomy device trials.^7,8^ It is being used as the primary outcome to test the success of novel thrombectomy techniques in 3 ongoing randomized clinical trials.^9,10,11^ Clinical evidence studies support first-pass recanalization as a sensitive primary end point for success of endovascular thrombectomy.^12,13,14^

Tirofiban is a glycoprotein IIb/IIIa receptor antagonist that inhibits platelet aggregation. The antiplatelet mechanism helps to reduce the early reocclusion following mechanical thrombectomy, which can then reduce the need for multiple passes.^15^ A recent randomized clinical trial, the Endovascular Treatment With vs Without Tirofiban for Patients With Large Vessel Occlusion Stroke (RESCUE BT) trial,^16^ tested the efficacy of tirofiban as an adjunctive treatment before endovascular treatment. Although the trial did not show therapeutic benefit of tirofiban on 90-day functional outcomes, a post hoc analysis of the trial reported an increase of the first-pass recanalization with tirofiban treatment compared with placebo.^17^ Nevertheless, the potential benefit of tirofiban in first-pass recanalization remains uncertain and needs to be evaluated as the primary outcome in a randomized clinical trial. This phase 2 randomized clinical trial aimed to test the hypothesis that intravenous tirofiban initiated before thrombectomy increases the likelihood of first-pass recanalization without increasing symptomatic intracranial hemorrhage in patients undergoing endovascular thrombectomy for large vessel occlusion who had an acute ischemic stroke.

## Methods

### Study Design

The OPTIMISTIC (One Pass Tirofiban in Management of Ischemic Stroke Thrombectomy in China) trial was a multicenter, prospective, open-label, blinded, end point phase 2 randomized clinical trial comparing intravenous tirofiban with usual care, without the use of intravenous or intraarterial antiplatelet agents prior to the first thrombectomy attempt. This study was conducted across 7 hospital stroke centers in China (Shanghai East Hospital, Shanghai Seventh People’s Hospital, Zhangzhou Municipal Hospital, Jinan Central Hospital, Shanghai Sixth People’s Hospital, Xuchang Central Hospital, and The First Affiliated Hospital of Ningbo University), with central ethics approval from the human research ethics committee of Shanghai East Hospital, Tongji University, and from each participating site. Written informed consent was obtained from patients or their surrogates prior to study enrollment. The trial was monitored by an independent data and safety monitoring board; the trial protocol and statistical analysis plan (finalized prior to the study data lock) are detailed in Supplement 1. This study followed the Consolidated Standards of Reporting Trials (CONSORT) reporting guideline.

### Recruitment

The total patients considered for endovascular thrombectomy were screened across 7 sites between April 30, 2021, and July 16, 2023. The study timeline is summarized in eFigure 1 in Supplement 2.

### Inclusion and Exclusion Criteria

Patients who had acute ischemic stroke, presenting to the participating hospitals for potential endovascular thrombectomy, were screened for the study. Study candidates were patients aged 18 to 85 years presenting with ischemic stroke within 24 hours from onset or last known well time with a clinical severity of 6 or more, measured by the National Institutes of Health Stroke Scale (NIHSS; scores range from 0 to 42, with higher scores indicating greater neurologic deficit), and occlusion of the internal carotid artery (ICA) or the M1 or M2 segment of the middle cerebral artery confirmed by computed tomography (CT) angiography or magnetic resonance imaging angiography. The M1 segment is the main trunk of the middle cerebral artery, and the M2 segment is the first-order branch of the main trunk of the middle cerebral artery. Patients were further selected by perfusion imaging with the following criteria required, according to the EXTEND-IA (Extending the Time for Thrombolysis in Emergency Neurological Deficits—Intra-Arterial) trial^18^: ischemic penumbra (ischemic penumbra represents ischemic brain tissue at risk of progressing to infarction but still salvageable with blood restoration) more than 10 mL; infarct core (the tissue that has already infarcted) less than 70 mL; and mismatch ratio (calculated by the total volume of penumbra and infarct core divided by infarct core volume) more than 1.2.

This study excluded patients with a history of atrial fibrillation, the main cause of cardioembolic stroke, because cardioembolic stroke is associated with high risk of symptomatic intracranial hemorrhage with tirofiban treatment.^19^ Patients treated with intravenous thrombolysis after stroke onset were also excluded. Patient selection criteria are detailed in the protocol in Supplement 1.

### Randomization and Masking

Patients who met the inclusion and exclusion criteria were assigned 1:1 to the tirofiban group and control group using permuted block randomization (randomly selected block size of 4 or 6) stratified by participating sites. A web-based, computer-generated randomization procedure was used. Treatment allocation was open label, as masking health personnel and patients during the acute phase of treatment would not be possible or practical. End point assessments were blinded. Neuroimaging data for each patient were uploaded to a central server, allowing an independent imaging analysis group to assess the primary outcome. Each patient’s imaging data were reviewed by 2 independent reviewers (Y.X. and H.S.), with disagreement resolved by consensus or a third party. All imaging analysis group members were vascular neurologists blinded to treatment allocation. The central reviewers (Y.X. and H.S.) were also blinded to site proceduralist reports on angiographic results after thrombectomy procedures (κ = 0.67 for first-pass recanalization assessment between central reviewers and site reports). The central reviewers determined the first attempt of the thrombectomy procedure according to the time stamps of digital subtraction angiography data (κ = 0.92 for first-pass recanalization assessment between the 2 central reviewers). A central imaging coordinator cross-checked with site proceduralist reports to confirm that the number of passes recorded in the reports was the same as the number of passes in the imaging database. Follow-up symptom severity (using the NIHSS) was assessed by local staff who were blinded to treatment allocation. The 90-day clinical follow-up (modified Rankin Scale [mRS], in which scores range from 0 to 6, with lower scores indicating less disability) was performed by a trained assessor from the leading study site who was also blinded to treatment allocation. A list of the study group and trial investigators is summarized in the eAppendix in Supplement 2.

### Procedures

Patients randomized to the intervention group received a bolus of 10 μg/kg of tirofiban, followed by an infusion of 0.1 μg/kg per minute for 24 hours. Tirofiban treatment was initiated immediately after randomization and prior to femoral puncture. Subsequent endovascular thrombectomy was performed according to standard local practice, although additional antiplatelet medications were not used during the tirofiban infusion period. The tirofiban regimen, including 24-hour infusion, was used according to the RESCUE BT trial.^16^

Patients randomized to the control group received standard preparation for endovascular thrombectomy, although intravenous or intraarterial antiplatelet medications were withheld prior to the first thrombectomy attempt. If residual stenosis was demonstrated, or reocclusion occurred after thrombectomy, patients could receive rescue therapy in accordance with local guidelines, including stent, angioplasty, or intraarterial tirofiban, if indicated. In the control group, intravenous tirofiban infusion was permitted after angioplasty or stent placement if patients were found to have large artery atherosclerosis as the underlying etiology.

### Outcomes

The predefined primary outcome was first-pass recanalization without symptomatic intracranial hemorrhage. First-pass recanalization was measured on digital subtraction angiography as a modified treatment in cerebral ischemia score of 2b or more (ranging from 0 to 3, in which 2b indicates substantial recanalization; 3, complete recanalization) in the target vessel after a single pass of the thrombectomy, before any other endovascular treatments including stenting, angioplasty, or intraarterial antiplatelet medications. Symptomatic intracranial hemorrhage was defined according to the Safe Implementation of Thrombolysis in Stroke-Monitoring Study criteria: type 2 parenchymal hematoma on follow-up noncontrast CT, 24 to 72 hours after randomization, combined with a neurological deterioration of 4 or more NIHSS points from baseline or leading to death within 72 hours.^20^

Secondary outcomes were recanalization after thrombectomy before any rescue treatment, recanalization at the end of all endovascular procedures, the number of thrombectomy passes, ongoing patency of targeted vessels at 24 to 72 hours, and 90-day functional outcomes. Recanalization was defined by a modified treatment in cerebral ischemia score of 2b or more. Ongoing vessel patency was defined by the arterial occlusive lesion recanalization score of 2 to 3, ranging from 0 to 3, in which a higher score indicates better recanalization. Functional outcomes at 90 days were assessed by the mRS, including the ordinal distribution of the mRS and the dichotomized outcome of functional independence (mRS 0 to 2 vs mRS 3 to 6). The safety outcomes included symptomatic intracranial hemorrhage within 72 hours and mortality within 90 days.

### Sample Size

The sample size was estimated using data from a retrospective analysis of the International Stroke Perfusion Imaging Registry (INSPIRE).^21^ In the INSPIRE analysis, 94 of 132 patients who received intravenous tirofiban before thrombectomy (71%) achieved first-pass recanalization without symptomatic intracranial hemorrhage compared with 55 of 115 patients who did not receive tirofiban (48%). Recruiting 200 patients (100 per arm) was estimated to yield 80% power to detect a treatment effect based on the proportion of participants with a primary outcome of 70% in the treatment group and 50% in the control group, using 2-sided α = .05, with an estimated 7% dropout rate.

### Statistical Analysis

The primary outcome analysis was conducted in the intention-to-treat population and in the per-protocol population. The intention-to-treat population included all patients who were randomly assigned to a trial group. For the intention-to-treat analysis, missing primary outcome data were assumed to be missing at random, and complete case analysis was performed with a sensitivity analysis to explore the effect of departures from the assumption of missing at random using a pattern-mixture model (detailed in the statistical analysis plan in Supplement 1).^22^ A post hoc analysis was conducted in the intention-to-treat population with missing data imputed by multivariate imputation by chained equations. Patients who did not have major protocol deviations were included in the per-protocol analysis as prespecified in the statistical analysis plan (Supplement 1). Safety outcomes were further analyzed on patients who received the assigned treatment.

The primary outcome was analyzed by modified Poisson regression with robust error estimation as prespecified in the statistical analysis plan (Supplement 1), adjusting for time from onset or last known well time to randomization (0 to 9 hours vs 9 to 24 hours) and vessel occlusion site (ICA vs other). The modified Poisson regression was used to analyze the secondary outcomes of recanalization assessed after thrombectomy (adjusted by time to randomization and occlusion site), at the end of all endovascular treatment (adjusted by time to randomization, occlusion site, and rescue treatment), and at 24 to 72 hours (adjusted by time to randomization, occlusion site, and rescue treatment). The median number of thrombectomy passes for each group was compared using median regression adjusted for time to randomization and vessel occlusion site. The differences in distribution of the mRS scores across the full scale at 90 days were assessed using ordinal logistic regression adjusted for age, time to randomization, and baseline NIHSS. Functional independence (mRS ≤2) at 90 days and mortality at 90 days were assessed using the modified Poisson regression adjusted for the same covariates as the ordinal mRS model. Although the statistical analysis plan prespecified unadjusted analysis of symptomatic intracranial hemorrhage with modified Poisson regression, due to very low observed counts of symptomatic intracranial hemorrhage, the rates were analyzed by estimating the risk difference with the respective 95% CI. Post hoc logistic regression analyses were conducted for the primary outcome and binary secondary outcomes.

Subgroup analyses were conducted as prespecified in the statistical analysis plan (Supplement 1). All statistical analyses were performed on Stata, version 13.0 (StataCorp LLC). Estimates of treatment effect are presented with 95% CIs. The statistical analysis plan did not prespecify correction for multiple comparisons for secondary outcomes or the subgroup analyses; thus, the reported CIs should not be used for hypothesis testing. A 2-sided *P* < .05 was considered statistically significant.

## Results

### Baseline Characteristics

**Figure 1.**
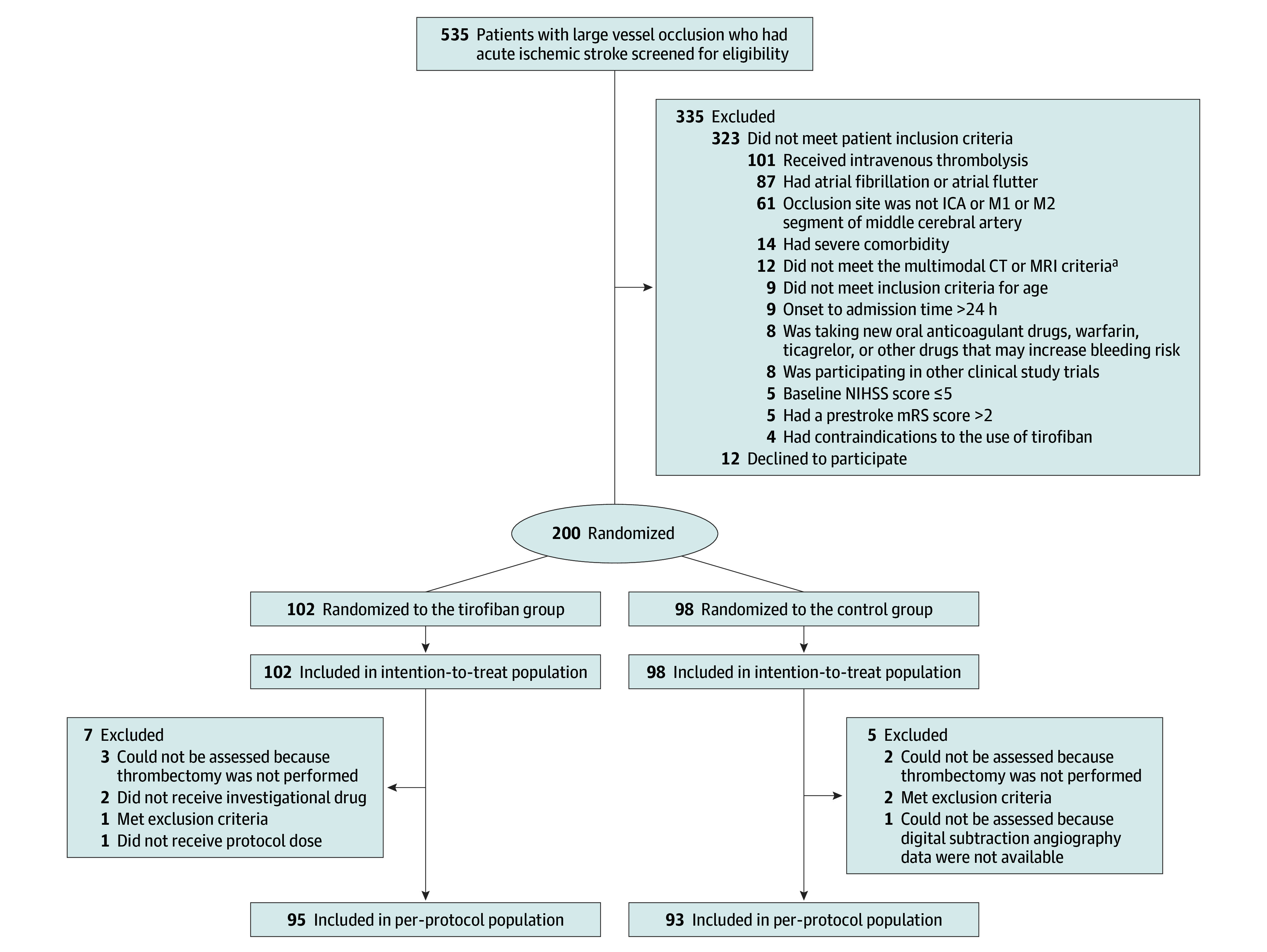
Patient Flow Diagram for the One Pass Tirofiban in Management of Ischemic Stroke Thrombectomy in China (OPTIMISTIC) Randomized Clinical Trial CT indicates computed tomography; ICA, internal carotid artery; MRI, magnetic resonance imaging; mRS, modified Rankin Scale (scores range from 0 to 6, with lower scores indicating less disability); NIHSS, National Institutes of Health Stroke Scale (scores range from 0 to 42, with higher scores indicating greater neurologic deficit). ^a^Multimodal CT or MRI criteria: ischemic penumbra, more than 10 mL; infarct core, less than 70 mL; and mismatch ratio, more than 1.2.

Among 535 patients who were considered and screened for endovascular thrombectomy, 200 were randomized (median age, 66 years [IQR, 58-72 years]; 54 female [27%] and 146 male [73%]). Of these patients, 102 were assigned to the tirofiban group, and 98 were assigned to the control group (Figure 1). No patients withdrew consent after randomization. Among the 200 patients included in the intention-to-treat analysis, 6 had a missing primary outcome, since 3 patients from each group did not have thrombectomy performed or recorded as planned (Figure 1). For the per-protocol analysis, 12 participants with major protocol deviations were excluded, 7 in the tirofiban group and 5 in the control group (detailed in eTable 1 in Supplement 2). For the safety analysis set, 2 patients were randomized to the tirofiban group but did not receive the assigned treatment; therefore, they were crossed over to the control group.

The baseline characteristics of the tirofiban and control groups are summarized in Table 1. The median time from stroke onset to groin puncture was 720 minutes (IQR, 446-935 minutes) for the tirofiban group and 718 minutes (IQR, 464-984 minutes) for the control group. There was a numerically lower proportion of patients with ICA occlusion in the tirofiban group compared with the control group (17% vs 25%). The proportion of patients in the tirofiban vs control group was higher for M1 occlusion (62% vs 55%) and for M2 occlusion (10% vs 9%). For the first-line thrombectomy device, the proportion of patients receiving the combined stent retriever and aspiration was 69% for the tirofiban and 65% for the control group. The proportion of patients who received rescue treatment for angioplasty was 56% in the tirofiban group and 61% in the control group; 36% of patients received rescue treatment for stenting in the tirofiban group compared with 39% in the control group. In the control group, 46% of patients received post-thrombectomy intraarterial tirofiban. For the study group, the median time from intravenous tirofiban bolus to the first pass was 62 minutes (IQR, 42-80 minutes), and the median time from groin puncture to the first-pass attempt was 40 minutes (IQR, 30-57 minutes). Details of tirofiban treatment are summarized in eTable 2 in Supplement 2. Additional procedural characteristics are summarized in eTable 3 in Supplement 2. The first-pass thrombectomy procedures are summarized in eFigure 2 in Supplement 2.

**Table 1.** Baseline Patient Characteristics and Procedural Characteristics of Intention-to-Treat Population

Characteristic	Patient group (N = 200)	
	Control (n = 98)	Tirofiban (n = 102)
Age, median (IQR), y	67 (58-71)	65 (57-73)
Sex, No./total No. (%)		
Female	25/98 (25)	29/102 (28)
Male	73/98 (75)	73/102 (72)
NIHSS at baseline, median (IQR)^a^	11 (8-16)	12 (8-15)
Premorbid mRS, No./total No. (%)^b^		
0	93/98 (95)	92/102 (90)
1	4/98 (4)	7/102 (7)
2	1/98 (1)	3/102 (3)
Medical history, No./total No. (%)		
Hypertension	59/98 (60)	47/102 (46)
Diabetes	20/98 (20)	19/102 (19)
Ischemic stroke	11/98 (11)	22/102 (22)
Hemorrhagic stroke	2/98 (2)	2/102 (2)
Ischemic heart disease	5/98 (5)	7/102 (7)
Hypercholesterolemia	4/98 (4)	4 /102 (4)
Smoking	28/98 (29)	28/102 (27)
Alcohol use	19/98 (19)	14/102 (14)
Baseline antiplatelet medication use, No./total No. (%)		
Aspirin	8/98 (8)	11/102 (11)
Aspirin and clopidogrel	3/98 (3)	0/102 (0)
Clopidogrel	0/98 (0)	1/102 (1)
Stroke etiology, No./total No. (%)		
Large artery atherosclerosis	83/98 (85)	82/102 (80)
Cardioembolism	8/98 (8)	8/102 (8)
Other or undetermined etiology	7/98 (7)	12/102 (12)
Workflow times, median (IQR), min		
Stroke onset or last known well to hospital arrival	536 (291-804)	524 (295-798)
Stroke onset or last known well to groin puncture	718 (464-984)	720 (446-935)
Hospital arrival to randomization	101 (72-138)	107 (77-160)
Groin puncture to first pass attempt^c^	41 (28-62)	40 (30-57)
Stroke onset or last known well to intravenous tirofiban bolus administration before thrombectomy^d^	NA	706 (432-938)
Intravenous tirofiban bolus to first pass attempt^e^	NA	62 (42-80)
Occlusion site, No./total No. (%)^f^		
Internal carotid artery	24/97 (25)	17/102 (17)
Middle cerebral artery M1	53/97 (55)	63/102 (62)
Middle cerebral artery M2	9/97 (9)	10/102 (10)
Tandem occlusion	11/97 (11)	10/102 (10)
Anterior cerebral artery	0/97 (0)	2/102 (2)
Perfusion imaging, median (IQR), mL		
Infarct core volume	11 (3-22)	11 (4-25)
Ischemic penumbra volume	105 (56-141)	84 (53-115)
Mismatch ratio	10 (6-28)	9 (5-21)
First-line thrombectomy procedure, No./total No. (%)^c^		
Stent retriever	4/95 (4)	5/99 (5)
Combined stent retriever and aspiration	62/95 (65)	68/99 (69)
Aspiration	18/95 (19)	16/99 (16)
Others	11/95 (12)	10/99 (10)
Rescue endovascular procedure, No./total No. (%)		
Angioplasty^f^	59/97 (61)	57/102 (56)
Stenting^f^	38/97 (39)	37/102 (36)
Intraarterial tirofiban	45/98 (46)	8/102 (8)

Abbreviations: mRS, modified Rankin Scale; NA, not applicable; NIHSS, National Institutes of Health Stroke Scale.

^a^ Scores range from 0 to 42, with higher scores indicating greater neurologic deficit.

^b^ Scores range from 0 to 6, with lower scores indicating less disability.

^c^ Data were not available for 6 patients; thrombectomy was not performed on 5 patients (3 in the tirofiban group and 2 in the control group), and digital subtraction angiography (DSA) data were not available for 1 patient in the control group.

^d^ Data were not available for 2 patients in the tirofiban group; the 2 patients were randomized to the tirofiban group but did not receive the assigned treatment.

^e^ Data were not available for 5 patients in the tirofiban group; 2 patients were randomized to the tirofiban group but did not receive the assigned treatment, and thrombectomy was not performed on 3 patients in the tirofiban group.

^f^ DSA data were not available for 1 patient in the control group.

### Primary Outcome

In the intention-to-treat analysis, 64 of the 99 patients receiving tirofiban (65%) and 48 of the 95 control patients (51%) demonstrated first-pass recanalization. Only 2 patients who demonstrated first-pass recanalization experienced symptomatic intracranial hemorrhage, both in the control group. The proportion of participants with a positive primary outcome rate was 65% (64 of 99) in the tirofiban group and 48% (46 of 95) in the control group (adjusted risk ratio [ARR], 1.34 [95% CI, 1.04-1.73]; *P* = .03) (Table 2). The results remained significant in the sensitivity analysis conducted under a range of assumptions about the missing data (lower-limit 95% CI of ARR remaining >1) (eFigure 3 in Supplement 2) and in the post hoc analysis with missing data imputed (ARR, 1.33 [95% CI, 1.03-1.71]; *P* = .03). The per-protocol analysis also showed an increased probability of the positive primary outcome in the tirofiban group (67% [64 of 95 patients]) compared with the control group (48% [45 of 93 patients]) (ARR, 1.40 [95% CI, 1.08-1.80]; *P* = .01), demonstrating the primary outcome. Results of post hoc logistic regression analyses are summarized in eTable 4 in Supplement 2.

**Table 2.** Primary and Secondary Outcomes of the Intention-to-Treat Population

Outcome	Patient group (N = 200)		Effect size (95% CI)	*P* value
	Control (n = 98)	Tirofiban (n = 102)		
Primary outcome				
First-pass recanalization without symptomatic intracranial hemorrhage, No./total No. (%)^a^	46/95 (48)	64/99 (65)	ARR, 1.34 (1.04 to 1.73)^b^	.03
Secondary efficacy outcomes				
First-pass recanalization, No./total No. (%)^a^	48/95 (51)	64/99 (65)	ARR, 1.29 (1.01 to 1.65)^b^	NA
Recanalization after thrombectomy, No./total No. (%)^a^	76/95 (80)	89/99(90)	ARR, 1.12 (1.00 to 1.27)^b^	NA
No. of thrombectomy passes, median (IQR)^a^	1 (1-2)	1 (1-2)	Adjusted coefficient, 0 (−0.42 to 0.42)^b^	NA
Recanalization at the end of the endovascular procedure, No./total No. (%)^c^	89/97 (92)	96/102 (94)	ARR, 1.02 (0.95 to 1.10)^d^	NA
Recanalization at 24-72 h, No./total No. (%)^e^	62/69 (90)	65/70 (93)	ARR, 1.04 (0.94 to 1.14)^d^	NA
90-d mRS 0-2, No./total No. (%)^f^^,^^g^	60/98 (61)	53/101 (53)	ARR, 0.85 (0.68 to 1.07)^h^	NA
90-d Ordinal mRS, median (IQR)^f^^,^^g^	2 (1-4)	2 (1-4)	Adjusted common OR, 0.67 (0.41 to 1.11)^h^	NA
Safety outcomes				
Symptomatic intracranial hemorrhage, No./total No. (%)^i^	6/98 (6)	0/101 (0)	Unadjusted risk difference, −0.06 (−0.11 to −0.01)^j^	NA
Mortality at 90 d, No./total No. (%)^f^	11/98 (11)	13/101 (13)	ARR, 1.16 (0.56 to 2.42)^h^	NA

Abbreviations: ARR, adjusted risk ratio; mRS, modified Rankin Scale; NA, not applicable; OR, odds ratio.

^a^ Data were not available for 6 patients; thrombectomy was not performed on 5 patients (3 in the tirofiban group and 2 in the control group), and digital subtraction angiography data were not available for 1 patient in the control group.

^b^ Treatment effect was adjusted for time from onset or last known well to randomization and occlusion site.

^c^ Digital subtraction angiography data were not available for 1 patient in the control group.

^d^ Treatment effect was adjusted for time from onset or last known well to randomization, occlusion site, and rescue treatment.

^e^ Data for follow-up computed tomography (CT) or magnetic resonance angiography were not available for 61 patients (32 in the tirofiban group and 29 in the control group).

^f^ One patient was lost to follow-up at 90 days in the tirofiban group.

^g^ Scores for mRS range from 0 to 6, with lower scores indicating less disability.

^h^ Treatment effect was adjusted for age, time from onset or last known well to randomization, and baseline National Institutes of Health Stroke Scale.

^i^ Data for follow-up noncontrast CT were not available for 1 patient in the tirofiban group.

^j^ Unadjusted risk difference was reported; relative risk was not measurable since the symptomatic intracranial hemorrhage rate was 0 in the tirofiban group.

### Secondary Outcomes

Secondary outcomes and safety outcomes are shown in Table 2 (intention-to-treat population) and in eTable 5 in Supplement 2 (per-protocol population). In the intention-to-treat population, the median number of thrombectomy passes was 1 (IQR, 1-2) in both groups; recanalization after thrombectomy alone was achieved in 76 of 95 control patients (80%) and in 89 of 99 patients in the tirofiban group (90%) (ARR, 1.12 [95% CI, 1.00-1.27]). At 90 days, 60 of 98 patients in the control group (61%) demonstrated functional independence compared with 53 of 101 patients in the tirofiban group (53%) (ARR, 0.85 [95% CI, 0.68-1.07]), while 11 of 98 control patients (11%) and 13 of 101 patients in the tirofiban group (13%) had died (ARR, 1.16 [95% CI, 0.56-2.42]).

### Safety Outcomes

In the intention-to-treat population, the symptomatic intracranial hemorrhage rate was 0% (0 of 101 patients) in the tirofiban group compared with 6% (6 of 98 patients) in the control group (unadjusted risk difference, −0.06 [95% CI, −0.11 to −0.01]). Two patients experienced type 2 parenchymal hematoma in the tirofiban group (2%) and 7 in the control group (7%). The rate of any intracranial hemorrhage was 23% in the tirofiban group and 22% in the control group. Serious adverse events are summarized in eTable 6 in Supplement 2. eTables 7 and 8 in Supplement 2 summarize the safety outcomes in the safety analysis set.

### Subgroup Analysis

**Figure 2.**
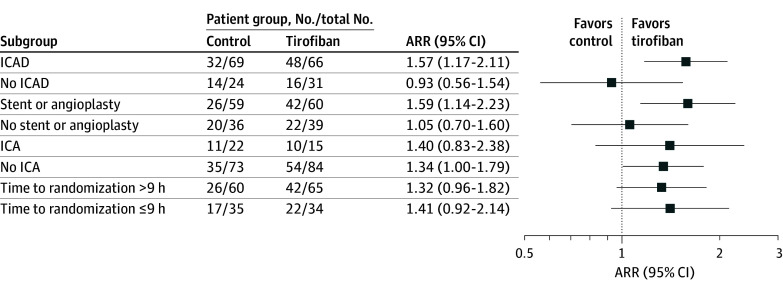
Forest Plot of the Primary Outcome Stratified by Prespecified Subgroups in the Intention-to-Treat Population Primary outcome data were not available for 6 patients (3 in the tirofiban group and 3 in the control group), and intracranial atherosclerotic disease (ICAD) classification was not available for an additional 4 patients without residual stenosis information (2 in the tirofiban group and 2 in the control group). ARR indicates adjusted risk ratio; ICA, internal carotid artery.

In the prespecified subgroup analysis, statistically significant heterogeneity of the treatment effect for the primary outcome was observed across subgroups with and without intracranial atherosclerotic disease (Figure 2). In the intracranial atherosclerotic disease subgroup, 73% of patients receiving tirofiban (48 of 66) and 46% of control patients (32 of 69) demonstrated the primary outcome of first-pass recanalization without symptomatic intracranial hemorrhage (ARR, 1.57 [95% CI, 1.17-2.11]). In the subgroup without intracranial atherosclerotic disease, the primary outcome rate was 52% (16 of 31 patients) in the tirofiban group and 58% (14 of 24 patients) in the control group (ARR, 0.93 [95% CI, 0.56-1.54]). In the subgroup without intracranial atherosclerotic disease, patients receiving tirofiban had a lower rate of 90-day functional independence (42%) compared with control patients (71%) (ARR, 0.57 [95% CI, 0.37-0.90]), while in the intracranial atherosclerotic disease subgroup, patients in both groups had similar rates of 90-day functional independence (58% vs 59%; ARR, 0.98 [95% CI, 0.75-1.28]).

## Discussion

In this multicenter randomized clinical trial, intravenous tirofiban administered before endovascular thrombectomy increased the likelihood of first-pass recanalization without symptomatic intracranial hemorrhage in patients who had acute ischemic stroke with no history of atrial fibrillation and no prior intravenous thrombolysis. The findings of this study are consistent with those from a post hoc analysis of the RESCUE BT trial^17^ showing an increase of first-pass recanalization in the tirofiban group compared with placebo (30.5% vs 23.5%; ARR, 1.24 [95% CI, 1.01-1.51]). Compared with the RESCUE BT trial, our study reports a higher rate of first-pass recanalization from endovascular thrombectomy. The first-pass recanalization rate in our study (51% in the control group) is consistent with a meta-analysis of 67 cohort studies that report an overall rate of first-pass recanalization at 45% when the recanalization was defined by a modified treatment in cerebral ischemia score of 2b to 3.^23^ The difference in our study and the RESCUE BT trial might be explained by the choice of a first-line endovascular thrombectomy device. The combined stent retriever and aspiration were chosen as a first-line device in over 60% of patients in our trial and in only in 22% of patients in the RESCUE BT trial. The combination of a stent retriever and aspiration device has been reported to be superior to a stent retriever alone in achieving first-pass recanalization.^17^

The findings of our study suggest that tirofiban may be a safe adjunct to endovascular treatment for patients who have had acute ischemic stroke. Symptomatic hemorrhagic transformation is the major safety concern after endovascular treatment in large vessel occlusion stroke. In patients receiving tirofiban as adjunctive treatment to thrombectomy, no symptomatic intracranial hemorrhage event was identified in our study. This may be related to the increase of the first-pass recanalization rate by tirofiban. A successful single pass of thrombectomy has been reported to reduce the hemorrhagic transformation rate compared with multiple passes, since it avoids repeated device-pass attempts that can lead to increased endothelial damage and blood-brain barrier disruption.^24^ A post hoc analysis of the RESCUE BT trial reported that patients who had cardioembolic stroke were at significantly higher risk of symptomatic intracranial hemorrhage with tirofiban treatment compared with placebo.^19^ Thus, the low bleeding risk of tirofiban in our study may be explained by excluding patients with a history of atrial fibrillation, the most common cause of cardioembolic stroke.^25^ The low bleeding risk may also be related to excluding patients who had received intravenous thrombolysis prior to thrombectomy in the study.

It should be noted that this phase 2 randomized clinical trial was not powered to draw conclusions on the efficacy of tirofiban treatment on 90-day functional outcomes of patients who have had a stroke. Our findings suggest an increase in the likelihood of less functional independence with tirofiban treatment, which might be explained by the futile and potentially harmful treatment effect of tirofiban in patients without intracranial atherosclerotic disease. The subgroup analysis raises the possibility that patients with intracranial atherosclerotic disease may be more likely to benefit from tirofiban treatment. This is consistent with the post hoc analysis of the RESCUE BT trial, in which intravenous tirofiban before endovascular treatment was associated with improved 90-day functional independence in the subgroup of patients with intracranial atherosclerotic disease causing large vessel occlusion.^26^ Together, the 2 trials generate a strong hypothesis that adjunctive tirofiban treatment should target patients with large vessel occlusion who have had acute ischemic stroke due to intracranial atherosclerotic disease and merit a future confirmatory phase 3 trial confined to this population. The functional outcomes of our study might also be confounded by rescue tirofiban therapy administered to 46% of patients in the control group. This confounding factor was analyzed in the RESCUE BT trial that categorized patients in the placebo group who received rescue tirofiban treatment into the tirofiban group.^16^ However, such an as-treated analysis did not demonstrate the benefit of tirofiban in 90-day functional outcomes.

### Limitations

This study has several limitations. First, there was no placebo administered in the control group; therefore, the proceduralist or clinical team had knowledge of the treatment group assignment for these patients. Second, there was a numerically lower rate of ICA occlusion in the tirofiban group. Although the occlusion site was adjusted as a potential confounder in the trial, ICA occlusions are known to be associated with a greater burden of clot and more than 1 pass to recanalize the artery, and this imbalance may have favored the tirofiban group. Third, the type of thrombectomy procedure was not standardized in this study. The choice of a first-line thrombectomy procedure varied across sites. By stratifying participating sites in the randomization, the thrombectomy strategy was well balanced between the study and control groups. Fourth, patients were selected by CT perfusion in this study; therefore, its results may not be generalizable to patients with large vessel occlusion who are selected by noncontrast CT. Fifth, the efficacy of tirofiban in facilitating first-pass recanalization may be affected by procedure time. A pronounced trough in circulation levels occurs 30 minutes after the bolus of intravenous tirofiban.^27^ The prolonged first-pass attempt in this study is possibly due to the high prevalence of intracranial atherosclerotic disease that requires comprehensive angiographic assessment. Another limitation of this study is that all patients were enrolled from China, which limits the generalizability of the trial results to Asian populations with a high prevalence of intracranial atherosclerotic disease (which is, however, a large proportion of stroke globally).

## Conclusions

In this randomized clinical trial that included patients who had acute ischemic stroke with no history of atrial fibrillation and no prior intravenous thrombolysis, intravenous tirofiban delivered prior to endovascular thrombectomy increased the likelihood of rapid recanalization during endovascular thrombectomy, particularly among patients with intracranial atherosclerotic disease. The findings of this study suggest that neurointerventionalists may consider this pre-procedure antiplatelet treatment to facilitate endovascular thrombectomy.

## Notes

Supplement 1.
Trial Protocol and Statistical Analysis Plan

Supplement 2. **eAppendix.** List of Study Group and Trial Investigators **eTable 1.** List of Major Protocol Deviations in Intention-to-Treat Population **eTable 2.** Tirofiban Treatment Information in Intention-to-Treat Population **eTable 3.** Additional Procedural Characteristics of Intention-to-Treat Population **eTable 4.** Post Hoc Logistic Regression Analysis of Primary and Secondary Outcomes in Intention-to-Treat Population **eTable 5.** Outcomes of Per-Protocol Analysis **eTable 6.** Severe Adverse Events Summary of Intention-to-Treat Population **eTable 7.** Safety Outcomes of Safety Analysis Set **eTable 8.** Severe Adverse Events Summary of Safety Analysis Set **eFigure 1.** Study Timeline **eFigure 2.** Plot of First-Pass Thrombectomy Procedures Across Sites in Intention-to-Treat Population **eFigure 3.** Sensitivity Analysis With Missing Primary Outcome Data in Intention-to-Treat Population

Supplement 3.
Nonauthor Collaborators

Supplement 4.
Data Sharing Statement

## References

[1] Powers WJ, Rabinstein AA, Ackerson T, . Guidelines for the Early Management of Patients With Acute Ischemic Stroke: 2019 update to the 2018 Guidelines for the Early Management of Acute Ischemic Stroke: A Guideline for Healthcare Professionals From the American Heart Association/American Stroke Association. Stroke. 2019;50(12):e344-e418. doi:10.1161/STR.0000000000000211 31662037

[2] Goyal M, Menon BK, van Zwam WH, ; HERMES collaborators. Endovascular thrombectomy after large-vessel ischaemic stroke: a meta-analysis of individual patient data from five randomised trials. Lancet. 2016;387(10029):1723-1731. doi:10.1016/S0140-6736(16)00163-X 26898852

[3] Albers GW, Marks MP, Kemp S, ; DEFUSE 3 Investigators. Thrombectomy for stroke at 6 to 16 hours with selection by perfusion imaging. N Engl J Med. 2018;378(8):708-718. doi:10.1056/NEJMoa1713973 29364767 PMC6590673

[4] Nogueira RG, Jadhav AP, Haussen DC, ; DAWN Trial Investigators. Thrombectomy 6 to 24 hours after stroke with a mismatch between deficit and infarct. N Engl J Med. 2018;378(1):11-21. doi:10.1056/NEJMoa1706442 29129157

[5] Saver JL, Goyal M, van der Lugt A, ; HERMES Collaborators. Time to treatment with endovascular thrombectomy and outcomes from ischemic stroke: a meta-analysis. JAMA. 2016;316(12):1279-1288. doi:10.1001/jama.2016.13647 27673305

[6] Zaidat OO, Castonguay AC, Linfante I, . First pass effect: a new measure for stroke thrombectomy devices. Stroke. 2018;49(3):660-666. doi:10.1161/STROKEAHA.117.020315 29459390

[7] Lapergue B, Blanc R, Costalat V, ; ASTER2 Trial Investigators. Effect of thrombectomy with combined contact aspiration and stent retriever vs stent retriever alone on revascularization in patients with acute ischemic stroke and large vessel occlusion: the ASTER2 randomized clinical trial. JAMA. 2021;326(12):1158-1169. doi:10.1001/jama.2021.13827 34581737 PMC8479584

[8] Turk AS III, Siddiqui A, Fifi JT, . Aspiration thrombectomy versus stent retriever thrombectomy as first-line approach for large vessel occlusion (COMPASS): a multicentre, randomised, open label, blinded outcome, non-inferiority trial. Lancet. 2019;393(10175):998-1008. doi:10.1016/S0140-6736(19)30297-1 30860055

[9] Contact aspiration versus stent retriever for recanalisation of acute stroke patients with basilar artery occlusion: the posterior circulation ASTER randomized trial protocol. ClinicalTrials.gov identifier: NCT05320263. Updated March 1, 2023. Accessed April 3, 2024. https://clinicaltrials.gov/study/NCT05320263

[10] TWIN2WIN (double stent (DS-EVT) versus primary thrombectomy with one stent (SS-EVT)). ClinicalTrials.gov identifier: NCT05632458. Updated November 30, 2022. Accessed April 3, 2024. https://clinicaltrials.gov/study/NCT05632458

[11] The safety and efficacy of Embotrap in treating acute ischemic stroke patients. ClinicalTrials.gov identifier: NCT05667103. Updated November 13, 2023. Accessed April 3, 2024. https://clinicaltrials.gov/study/NCT05667103

[12] Gupta R. Response to: correspondence on “Technique and impact on first pass effect primary results of the ASSIST global registry” by Gupta *et al.* J Neurointerv Surg. 2024;17(1):e021839. 38969495 10.1136/jnis-2024-021839 PMC11672036

[13] Nguyen TN, Abdalkader M, Qureshi MM, . First-line stent retriever versus contact aspiration or combined technique for endovascular therapy of posterior cerebral artery occlusion stroke: the PLATO study. Stroke Vasc Interv Neurol. 2024;4(1):e001004. doi:10.1161/SVIN.123.001004 PMC12778457 41586053

[14] Jang KM, Choi HH, Nam TK, Byun JS. Clinical outcomes of first-pass effect after mechanical thrombectomy for acute ischemic stroke: a systematic review and meta-analysis. Clin Neurol Neurosurg. 2021;211:107030. doi:10.1016/j.clineuro.2021.107030 34823155

[15] Kang DH, Kim YW, Hwang YH, Park SP, Kim YS, Baik SK. Instant reocclusion following mechanical thrombectomy of in situ thromboocclusion and the role of low-dose intra-arterial tirofiban. Cerebrovasc Dis. 2014;37(5):350-355. doi:10.1159/000362435 24941966

[16] Qiu Z, Li F, Sang H, ; RESCUE BT Trial Investigators. Effect of intravenous tirofiban vs placebo before endovascular thrombectomy on functional outcomes in large vessel occlusion stroke: the RESCUE BT randomized clinical trial. JAMA. 2022;328(6):543-553. doi:10.1001/jama.2022.12584 35943471 PMC9364124

[17] Yuan J, Ge H, Tao Z, . Effect of intravenous tirofiban versus placebo on first-pass successful reperfusion in endovascular stroke thrombectomy: insights from the RESCUE BT randomized clinical trial. J Am Heart Assoc. 2024;13(21):e036350. doi:10.1161/JAHA.124.036350 39494598 PMC11935663

[18] Campbell BC, Mitchell PJ, Kleinig TJ, ; EXTEND-IA Investigators. Endovascular therapy for ischemic stroke with perfusion-imaging selection. N Engl J Med. 2015;372(11):1009-1018. doi:10.1056/NEJMoa1414792 25671797

[19] Rong B, Guo Z, Gao L, . Association of tirofiban treatment with outcomes following endovascular therapy in cardioembolic stroke: insights from the RESCUE BT randomized trial. Eur J Med Res. 2023;28(1):473. doi:10.1186/s40001-023-01406-x 37915101 PMC10621173

[20] Wahlgren N, Ahmed N, Dávalos A, ; SITS-MOST investigators. Thrombolysis with alteplase for acute ischaemic stroke in the Safe Implementation of Thrombolysis in Stroke-Monitoring Study (SITS-MOST): an observational study. Lancet. 2007;369(9558):275-282. doi:10.1016/S0140-6736(07)60149-4 17258667

[21] International Stroke Perfusion Imaging Registry (INSPIRE). Sydney Brain Center. Accessed January 14, 2021. https://inspire.brainimaginggroup.com/login/

[22] White IR, Horton NJ, Carpenter J, Pocock SJ. Strategy for intention to treat analysis in randomised trials with missing outcome data. BMJ. 2011;342:d40. doi:10.1136/bmj.d40 21300711 PMC3230114

[23] Abbasi M, Liu Y, Fitzgerald S, . Systematic review and meta-analysis of current rates of first pass effect by thrombectomy technique and associations with clinical outcomes. J Neurointerv Surg. 2021;13(3):212-216. doi:10.1136/neurintsurg-2020-016869 33441394 PMC9041815

[24] Luby M, Hsia AW, Nadareishvili Z, . Frequency of blood-brain barrier disruption post-endovascular therapy and multiple thrombectomy passes in acute ischemic stroke patients. Stroke. 2019;50(8):2241-2244. doi:10.1161/STROKEAHA.119.025914 31238832 PMC6646098

[25] Han SW, Nam HS, Kim SH, Lee JY, Lee KY, Heo JH. Frequency and significance of cardiac sources of embolism in the TOAST classification. Cerebrovasc Dis. 2007;24(5):463-468. doi:10.1159/000108438 17878729

[26] Sang H, Xie D, Tian Y, . Association of tirofiban with functional outcomes after thrombectomy in acute ischemic stroke due to intracranial atherosclerotic disease. Neurology. 2023;100(19):e1996-e2006. doi:10.1212/WNL.0000000000207194 36941074 PMC10186214

[27] Kimmelstiel C, Badar J, Covic L, . Pharmacodynamics and pharmacokinetics of the platelet GPIIb/IIIa inhibitor tirofiban in patients undergoing percutaneous coronary intervention: implications for adjustment of tirofiban and clopidogrel dosage. Thromb Res. 2005;116(1):55-66. doi:10.1016/j.thromres.2004.11.011 15850609

