# Supplementary material for: Tirofiban on First-Pass Recanalization in Acute Stroke Endovascular Thrombectomy: The OPTIMISTIC Randomized Clinical Trial
Source: JAMA Netw Open. 2025 Apr 17;8(4):e255308. doi: 10.1001/jamanetworkopen.2025.5308 (PMC12006867; doi:10.1001/jamanetworkopen.2025.5308)
Supplement: Supplement 1. — Trial Protocol and Statistical Analysis Plan [file jamanetwopen-e255308-s001.pdf]

One Pass Tirofiban In Management of Ischemic Stroke Thrombectomy In China (OPTIMISTIC)

Study protocol

Protocol Designer: Shanghai East Hospital, School of Medicine, Tongji University, Shanghai, China

Leading PI site: Shanghai East Hospital, School of Medicine, Tongji University, Shanghai, China

Study duration: 3 years

Version number: 3.1

Version date: September 5th, 2023

Contact information:

Address: Department of Neurology, No. 1800, Yuntai Road, Pudong New District, Shanghai

Tel: 021-38804518-22105

Email: ligang@tongji.edu.cn

Signature of principal investigator:

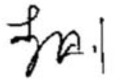

Date: September 5th, 2023

Contact information of principal investigator

Department of Neurology, Shanghai East Hospital, School of Medicine, Tongji University, Shanghai, China

Name: Gang Li	
Title: Professor, chief physician	
Address: No.1800, Yuntai Road, Pudong New District, Shanghai. 200127	
Telephone number (Office):	021-38804518-22107
Mobile phone:	13621691786
Email:	ligang@tongji.edu.cn

Contact information of investigator team members

Department of Neurology, Shanghai East Hospital, School of Medicine, Tongji University, Shanghai, China

Name: Yaping Xiao	
Title: attending physician	
Address: No.1800, Yuntai Road, Pudong New District, Shanghai	
Telephone number (Office)	021-38804518-22106
Mobile phone	13916980530
Email	Xiaoyaping2001@163.com

Department of Neurology, Shanghai East Hospital, School of Medicine, Tongji University, Shanghai, China

Name: Hao Shen	
Title: attending physician	
Address: No.1800, Yuntai Road, Pudong New District, Shanghai	
Telephone number (Office)	021-38804518-22106
Mobile phone	15900562941
Email	shenhao717@126.com

Department of Neurology, Shanghai East Hospital, School of Medicine, Tongji University, Shanghai, China

Name: Feifeng Liu	
Title: physician	
Address: No.1800, Yuntai Road, Pudong New District, Shanghai	
Telephone number (Office)	021-38804518-22106
Mobile phone	15121100573
Email	liufeifeng7@163.com

Department of Neurology, Shanghai East Hospital, School of Medicine, Tongji University, Shanghai, China

Name: Chen Chen	
Title: physician	
Address: No.1800, Yuntai Road, Pudong New District, Shanghai	
Telephone number (Office)	021-38804518-22106
Mobile phone	15921119641
Email	15921119641@163.com

I. Synopsis

Protocol title	Short title: One Pass Tirofiban In Management of Ischemic Stroke Thrombectomy In China (OPTIMISTIC)<br>Full title: Efficacy and safety of tirofiban in thrombectomy for acute ischemic stroke: a prospective, randomized, open label, endpoint blinded multicenter clinical study
Version number<br>Version date	V3.1<br>September 5th, 2023
Principal Investigator	Gang Li
Study objective	• Primary Objective<br>To determine whether intravenous tirofiban treatment can increase the probability of first-pass recanalization of targeted vessels without increasing the risk of symptomatic intracranial hemorrhage in acute ischemic stroke patients treated with thrombectomy.<br>• Secondary objectives<br>To determine whether intravenous tirofiban combined with thrombectomy, compared to thrombectomy alone, affects the following outcomes:<br>the recanalization rate of targeted vessels after thrombectomy, the recanalization rate of targeted vessels at the end of endovascular treatment, the recanalization rate of targeted vessels after 24-72 hours, symptomatic intracranial hemorrhage, and 90-day functional outcomes (dichotomized 90-day mRS, ordinal scale of 90-day mRS); death at 90 days; number of thrombectomy passes.
Study design	This study is a prospective, randomized, open-label, endpoint-blind study. Patients who meet the inclusion criteria are assigned 1:1 to the study group and control group according to the permuted-block randomization method to ensure the balance between and within the groups. Randomization is stratified by participating sites.
Study population	Acute ischemic stroke patients with large vessel occlusion
Inclusion/	Inclusion criteria

exclusion<br><br>Criteria	1. Age 18-85 years (inclusive);<br>2. Acute ischemic stroke within 24 hours of onset, and NIHSS >5 points.<br>3. Acute large vessel occlusion, including internal carotid artery, M1 or M2 segment of middle cerebral artery.<br>4. Multimodal CT/MRI imaging completed before thrombectomy, and demonstrating penumbra>10ml, infarct core volume <70ml, and mismatch rate >1.2;<br>5. Planned to receive endovascular treatment;<br>6. Informed consent was obtained.<br><br>Exclusion criteria<br><br>1. History of atrial fibrillation or atrial flutter, or 12-lead ECG before randomization and after admission showing atrial flutter or atrial fibrillation;<br>2. Treatment with intravenous thrombolysis for current stroke or currently taking direct oral anticoagulants (DOACs), warfarin, ticagrelor and other drugs that may increase the risk of bleeding.<br>3. Pre-stroke disability (pre-stroke mRS score > 2).<br>4. Severe comorbidity (such as severe cardiopulmonary dysfunction, or terminal cancer with expected survival less than 90 days).<br>5. Hypodensity lesion on CT covering more than 1/3 of the territory of the middle cerebral artery.<br>6. Allergy to tirofiban or any other contraindication to the use of tirofiban (active internal bleeding, history of intracranial hemorrhage, intracranial tumors, arteriovenous malformations, and intracranial

	aneurysms, history of thrombocytopenia from the use of tirofiban previously).<br>7. Contraindications to the contrast agents used in multimodal CT/MRI examination (such as allergy to the contrast agents, etc.).<br>8. Pregnant or breastfeeding women.<br>9. Patients currently participating in other clinical study trials.<br>10. Other conditions determined by the investigator to preclude the patient from inclusion in this study.
Study drug	Investigational drug:<br>Drug name: Tirofiban hydrochloride sodium chloride injection<br>Drug specifications: 100ml: Tirofiban hydrochloride 5mg
Drug regimen	The enrolled subjects are randomized to the following two groups in a 1:1 ratio:<br>**The study group**: intravenous tirofiban combined with thrombectomy. Tirofiban intravenous therapy is initiated as soon as possible after randomization and before femoral artery puncture. Tirofiban is used as follows: tirofiban 10 μg/kg, intravenous bolus injection within 3 minutes, and 0.1 μg/kg/min intravenous infusion at least for 24 hours. The site investigators decide on the subsequent antiplatelet treatment plan based on the patient's condition after 24 hours of intravenous infusion.<br>**The control group**: treated with routine thrombectomy. The control group is not allowed to use intravenous or intraarterial antiplatelet drugs before the first thrombectomy attempt.
Evaluation indicators	Image assessment:<br>1. Baseline: brain multimodal CT, including non-contrast CT, CTA, and CTP; If the patient has extracranial occlusion of the internal

	carotid artery, carotid artery CTA is required.<br>2. Treatment process and immediate outcome: DSA<br>3. 24-72 hours: brain non-contrast CT and brain CTA; If brain CTA cannot be acquired, brain MRA is acceptable; If the patient has extracranial occlusion of the internal carotid artery, it is necessary to acquire carotid artery CTA or MRA at the same time.<br>4. Evaluation of recanalization: mTICI grade on DSA; AOL grade on 24-72-hour CTA or MRA<br>5. Evaluation of symptomatic intracranial hemorrhage: SITS-MOST criteria.<br>Clinical assessment:<br>1. Baseline: NIHSS, GCS.<br>2. 24-72 hours (± 8 hours): NIHSS, GCS.<br>3. 90 days: mRS.

II. Contents

I. Synopsis 4

II. Contents 8

III. Administration information 11

Protocol revision history 11

3. Detailed patient withdraw criteria; 12

Study registration information 12

IV. Study background 13

1. epidemiology 13

2. The pathophysiological mechanism of thrombosis and antiplatelet drugs 14

3. Study status of tirofiban for endovascular treatment in acute ischemic stroke 16

4. Summary 18

V. Study objective 18

1. Primary objective 19

2. Secondary objectives 19

VI. Methods 19

1. Overall design 19

2. Study schema (flow chart) 20

3. Study population 20

4. Inclusion and exclusion criteria 21

4.1 Inclusion criteria 21

4.2 Exclusion criteria 21

5.1 Institutional ethics committee approval 22

5.2 Informed consent 23

6 Randomization 24

6.1 Randomization procedure 24

6.2 Blinded follow-up 25

6.3 Blinded review 25

7 Interventions 25

7.1 Study drug 25

7.2 Drug dosage and regimen 26
7.3 Drug management 26
7.4 Procedural method 26
7.5 Prohibited drugs in the study 27
8. Withdrawal and exclusion criteria 27
8.1 Withdrawal decided by the investigator 27
8.2 Withdrawal decided by the participant 28
8.3 Criteria of exclusion from data analysis 29
9. Study outcomes: 29
10. Data collection and follow-up 30
10.1 Patient Contact Details log 31
10.2 Randomization assessment 31
10.3 Baseline data 32
10.4 Treatment information (0-24h) 32
10.5 Follow-up data 33
10.6 Discontinuation of assigned treatment and protocol violation 34
10.7 Informed consent 35
10.8 Serious adverse events 35
VII. Study flow chart 35
VIII. Serious adverse events 36
1. Definition: 36
3. Monitoring of SAEs 37
4. Monitoring of SUSAR 38
IX. Quality assurance 38
1. Preservation and supervision of original data/documents 38
2. Preparation before the study 39
3. Site staff training 39
4. Study monitoring 40
5. Supervision and inspection by government regulators 40
6. Source data 40
7. Archiving of study documents 41

8. Study progress..41
X. Data management **41**
XI. Statistics **41**
1. statistical considerations..41
2. General principles..42
3. Analysis 42
4. Primary outcome and sample size estimation 42
XII. Publications and reports..**43**
XIII. Organization..**44**
XIV. Funding..**45**
XV. Timeline **45**
XVI. References **46**
XVII. appendix **52**
1. modified Rankin Scale (mRS) 52
2. National Institutes of Health Stroke Scale (NIHSS)..54
3. target artery recanalization grade (AOL grade): 63

III. Administration information

Protocol revision history

Version number	Version date	Revision summary
1.0	March 14, 2021	First version
2.0	May 24, 2021	1. Revised the inclusion criteria of stroke onset time from 4.5-24 hours to within 24 hours of onset;<br>2. Revised the exclusion criteria of pre-stroke mRS from pre-stroke mRS ≥ 2 to pre-stroke mRS > 2;<br>3. Added the following exclusion criteria: currently taking direct oral anticoagulant drugs (DOACs), warfarin, ticagrelor and other drugs that may increase the risk of bleeding;<br>4. Further detailed the study drug administration method and endovascular procedure approach;<br>5. Added the information of prohibited s drugs in this study;<br>6. Further detailed the definition of SAE;<br>7. added study registration information.

2.1	February 10, 2022	1. Removed the requirement that any intracranial hemorrhage transformation needs to be reported in SAE;<br>2. Revised the follow-up image time to 24-72 hour (± 8 hours);<br>3. Revised the inclusion criteria of age from 18-80 years to 18-85 years;<br>4. Extended the study completion time to December 2023.
3.0	May 18th 2023	1. Added antiplatelet therapy strategies after intravenous tirofiban infusion in the study group;<br>2. Detailed the definition of first-pass recanalization;<br>3. Detailed patient withdraw criteria;<br>4. Added study funding information.
3.1	September 5th 2023	1. Further clarified the definition of first-pass recanalization.<br>2. Added the registry data information used for sample size cacluation.3. Clarified that thrombectomy procedure was delivered according to local guidelines in the control group.

Study registration information

This study has been registered at clinicaltrials.gov (NCT04851457) and the China Clinical Trial Registry (ChiCTR 2100045661).

IV. Study background

1. epidemiology

Stroke is a worldwide health problem, with an estimated 13.7 million new strokes and approximately 5.8 million deaths annually (http://world-stroke.org/), making stroke the second leading cause of death in the world [1]. In addition, there are currently more than 80 million stroke survivors worldwide, of whom an estimated 50% suffer from moderate to severe neurologic deficits [2], and a quarter require permanent assistance. Stroke has become the third leading cause of adult disability worldwide [3]. In China, the number of new stroke cases is more than 2 million annually. What's worse, the burden of stroke is predicted to rise further in the coming decades as a result of population aging and ongoing high prevalence and inadequate management of risk factors such as hypertension, which is recognized as a major global public health challenge[4]. [1234]

Stroke can be classified into ischaemic stroke (occlusion of a blood vessel) or hemorrhagic stroke (bleeding into the brain), with the former making up about 70% of strokes[2]. Acute ischemic stroke (AIS) is a vascular accident caused by thrombosis or embolism that occludes a cerebral vessel supplying a specific region of the brain, resulting in ischemia and hypoxic necrosis of local brain tissue and corresponding neurological deficits. After an ischaemic stroke, there is a core area where damage to the brain is irreversible and an area of penumbra where the brain has lost function owing to decreased blood flow but is not irreversibly injured. Rapid reperfusion therapy aims to restore blood flow in the ischaemic penumbra and salvage neurons in the penumbra, returning them to normal function [5, 6]. Therefore, early recanalization therapy is of great significance in reducing post-stroke disability and has become the mainstay of AIS treatment.

In the past 20 years, intravenous use of recombinant tissue plasminogen activator (rt-PA) has become the main method to achieve vascular recanalization after ischemia [7,8].

However, due to the narrow therapeutic time window of rt-PA treatment, many contraindications to its use, and the low effectiveness of recanalization in the case of large artery occlusion, in addition to the risk of serious secondary bleeding [9], its clinical application has been limited to approximately 15% of patients with acute ischaemic stroke. In addition, arterial interventional therapy has demonstrated much higher efficacy in large artery occlusions [10]. A growing body evidence has shown that arterial interventional therapy can benefit some patients with ischemic stroke [11], and it has become the preferred treatment for patients with anterior circulation large vessel occlusion stroke within 24 hours, although screening with advanced imaging techniques is required to select patients who will benefit over this extended timeframe [12, 13]. However, arterial interventional therapy can cause endothelial injuries leading to local activation of platelet aggregation and subsequent thromboembolic complications or early re-occlusion [14, 15]. Overall, up to half of AIS patients still fail to achieve effective vascular reperfusion even after receiving rt-PA and arterial interventional therapy [16-18]. The reperfusion of occluded vessels and their downstream microcirculation is a very important factor in determining the clinical prognosis of AIS patients [19]. Therefore, it is particularly important to seek effective and safe therapy combined with rt-PA or arterial intervention for AIS patients.

2. The pathophysiological mechanism of thrombosis and antiplatelet drugs

Thrombosis is one of the key pathophysiological mechanisms of AIS, and platelet activation plays a central role in the process of arterial thrombosis. Thrombosis requires three elements: vascular endothelial cell injury, changes in blood flow, and increased blood coagulation. Exposure of Von Willebrand factor (VWF), a subendothelial matrix protein, and collagen occurs after rupture of atherosclerotic plaques, interventional therapy, or other causes of vascular endothelial cell injury [20]. VWF binds to naked collagen and unfolds it, exposing multiple binding sites of platelet glycoproteins (GPIb-IX-V). Under arterial shear stress, GPIb interacts with VWF to mediate the transient

adhesion of platelets [21], enabling GPVI on the surface of platelets to combine with collagen to achieve stable adhesion of platelets [22, 23]. Collagen binding to GPVI can lead to platelet activation and the release of soluble agonists adenosine diphosphate (ADP), thromboxane A2 (TXA2) and thrombin. The released ADP, TXA2 and thrombin can act on P2Y purine receptors 1 and 12 (P2Y1 and P2Y12)in addition to directly activating platelets [24-27].

Figure 1 Mechanism of platelet adhesion and aggregation [31] (Due to the copyright issue please find the figure on the page 2 in reference 31)

Activated platelets cause conformational changes of GP IIb/IIIa through "inward outward" signaling, which changes them from a low-affinity state at rest to a high-affinity state. Activated GPIIb/IIIa can bind soluble plasma proteins, including VWF, fibronectin and its main ligand fibrinogen, and promote stable platelet aggregation and thrombosis [28, 29]. The production of thrombin enhances the activation of platelets and activates the coagulation process, enabling the formation of a stable fibrin mesh [30] (Figure 1). Therefore, moderation of platelet activation pathways is a key focus of AIS prevention and treatment.

At present, four classes of antiplatelet drugs are mainly used in clinics (Figure 2): ① cyclooxygenase 1 (cox1) inhibitors, such as aspirin. Cox1 is the enzyme that catalyzes the generation of TXA2 from arachidonic acid. Aspirin prevents TXA2 production by irreversibly inhibiting cox1, thereby inhibiting TXA2 induced platelet activation and aggregation [32, 33]; ② P2Y12 receptor inhibitors such as cangrelor, clopidogrel, prasugrel and ticagrelor. The P2Y12 receptor plays an important role in mediating the sustained activation of GPIIb/IIIa, the major platelet adhesion receptor, in response to ADP stimulation. P2Y12 receptor antagonists inhibit ADP induced enhancement of platelet activation, thereby producing potent antithrombotic effects [34]. However, up to 10% of patients on dual antiplatelet therapy with aspirin and clopidogrel still experience recurrent ischemic events within 12 months. In addition, platelet reactivity

continues to increase in 30-40% of patients treated with clopidogrel [35]. The existence of these problems has inspired people to develop new antiplatelet drugs; ③ Protease activated receptor 1 (PAR1) antagonist, such as vorapaxar. Thrombin is the most potent platelet agonist. PAR1 antagonists target thrombin, thereby exerting antithrombotic effects [36]. However, several studies have shown that vorapaxar significantly increases the rate of intracranial hemorrhage in patients with ischemic stroke [37], limiting its application in clinical practice. This drug is currently contraindicated for patients with a history of stroke, transient ischemic attack or intracranial hemorrhage; ④ GPIIb / IIIa receptor antagonists (GPI), such as abciximab, tirofiban and eptifibatide. GpIIb/IIIa receptor antagonists target the final pathway of platelet aggregation by competing with fibrinogen and vWF for binding to GpIIb/IIIa receptors [38]. One additional antiplatelet drug that inhibits phosphodiesterase, cilostazol, is currently only approved by the US Food and Drug Administration (FDA) for the treatment of peripheral vascular disease.

3. Study status of tirofiban for endovascular treatment in acute ischemic stroke

Tirofiban is the main GPIIb/IIIa receptor antagonist used in China. Tirofiban, which was approved in China in 2004, has the characteristics of strong specificity, high, though dose-dependent potency, and reversibility [39]. It can act on the final pathway of platelet aggregation, effectively preventing platelet mediated thrombosis and vessel re-occlusion. The safety and efficacy of tirofiban in the treatment of patients with acute coronary syndrome (ACS) have been extensively confirmed, and it has been widely used in clinical practice [40-49].

Figure 2 Diagram of antiplatelet therapeutic targets [31] (Due to the copyright issue please find the figure on the page 4 in reference 31)

In recent years, the application of tirofiban in AIS has attracted much attention.

Investigators and clinicians from different countries are focused on the application of tirofiban and have conducted a series of clinical trials to explore the effectiveness and safety of tirofiban in preventing early arterial re-occlusion and thromboembolic complications in AIS [50-74]. However, due to the limited sample size of most studies and the heterogeneity of treatment protocols and dosage regimens, the results of the studies thus far are contradictory and inconclusive. A consensus on the safety and efficacy profiles of tirofiban in AIS has not yet been achieved from the meta-analysis and systematic review based on these clinical trials. Several studies including meta-analysis [14, 64] have concluded that the combined application of tirofiban and endovascular treatment (EVT) of ischaemic stroke will increase the risk of intracranial hemorrhage (ICH). A prospective cohort study in 2013, which enrolled 191 consecutive patients who underwent EVT from 2006 to 2011 concluded that the use of tirofiban was associated with an increased risk of fatal ICH and a poor prognosis [14]. Moreover, a cohort study from Wu showed that the bleeding risk of tirofiban in EVT is dose-dependent [64]. However, several other meta-analyses have reached the opposite conclusion. Guo et al [75] found that early use of tirofiban treatment did not increase the risk of any ICH and death in AIS patients receiving EVT, but there was no association between tirofiban treatment and functional outcome or recanalization rate, suggesting that tirofiban treatment may be safe for AIS patients, but further study is needed to test its efficacy. Meanwhile, the study of Zhou et al [76] found that tirofiban treatment may be safe for AIS patients receiving intravenous thrombolysis, but its effect on improving functional outcomes is unclear. A recent meta-analysis [77] suggested that tirofiban treatment significantly increased the incidence of good functional outcomes and did not increase the risk and mortality or symptomatic intracranial hemorrhage in the Chinese population. This analysis included more recently published studies. In conclusion, those studies including the latest meta-analysis and review are limited and the sample sizes were small. Most of the included studies are observational studies rather than randomized controlled trials (RCTs), which may affect the results due to the related inherent risk of bias. At present, it is impossible to draw a conclusion on the efficacy of tirofiban for AIS patients receiving EVT. Therefore, a high-quality

multicenter randomized controlled trial with a large sample size is needed to provide reliable clinical evidence on the safety and efficacy of tirofiban in AIS.

4. Summary

Based on the efficient dose-dependent inhibitory effect of tirofiban on platelet aggregation [78] and conflicting conclusions from previous studies applying different routes of administration (particularly by intravenous and intra-arterial pathways) [51, 60, 64], it is hypothesized that therapy regimens of tirofiban (including the route of administration and medication dosage) perhaps account for the variation in clinical benefits and bleeding risks during EVT. We reviewed the studies on tirofiban dosage during endovascular treatment of acute large-artery occlusive stroke. Yang et al. [51] showed that when a large dose of 10 μg / kg of tirofiban was injected intra-arterially or intravenously, and then intravenously at a speed of 0.15 μ g / (kg · min) for 12-24 hours as an adjunct to EVT, intravenous tirofiban had a high recanalization rate and good prognosis, while intraarterial tirofiban has higher bleeding rate and mortality. Pan, Torgano, Gruber, et al [58, 62, 73] applied the same or greater dose of tirofiban intravenously, and the results suggested that the risk of intracranial hemorrhage and death was not increased. The safety of this dose of tirofiban in AIS patients was supported. According to the recommended usage of tirofiban in the instruction manual of angioplasty / intra-arterial atherectomy in ACS patients, we preliminarily concluded that for some patients, tirofiban 10 ug/kg intravenous bolus injection and 0.15ug/ (kg. min) intravenous infusion for 12-24 hours is clinically feasible. Therefore, we designed this prospective, randomized, controlled, open-label, multicenter clinical study and selected this dose of tirofiban to evaluate the safety and efficacy of tirofiban in combination with EVT in AIS patients and provide more basis for clinical study and clinical application.

V. Study objective

1. Primary objective

To determine, for acute ischemic stroke patients treated with thrombectomy, whether intravenous tirofiban treatment can increase the probability of first-pass recanalization of targeted vessels without increasing the risk of symptomatic intracranial hemorrhage.

2. Secondary objectives

To determine whether intravenous tirofiban combined with thrombectomy, compared to thrombectomy alone, has affects the following outcomes: the recanalization rate of targeted vessels after thrombectomy, the recanalization rate of targeted vessels at the end of endovascular treatment, the recanalization rate of targeted vessels at 24-72 hours, symptomatic intracranial hemorrhage, and 90-day functional outcomes(dichotomized 90-day mRS, ordinal scale of 90-day mRS); death at 90 days; number of thrombectomy passes.

VI. Methods

1. Overall design

This study is a multicenter, prospective, randomized, controlled, open-label, blinded-endpoint study. A total of 200 patients with acute ischemic stroke who are scheduled to undergo thrombectomy will be recruited from 10-15 hospitals in China. They will be randomly assigned 1:1 to the study group or control group. The study design is shown by the following study schema (study design flow chart).

2. Study schema (flow chart)

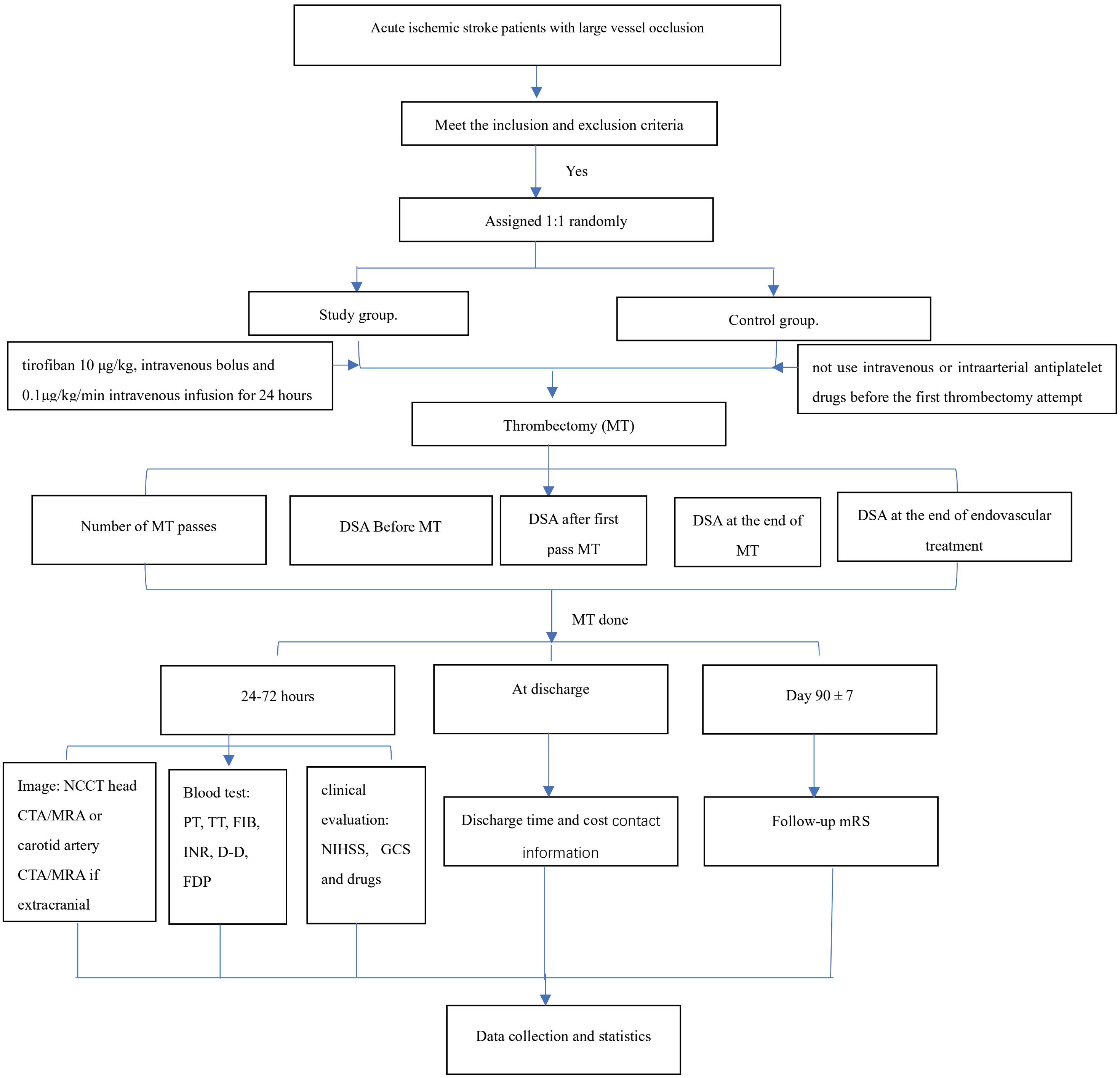

3. Study population

All patients with acute ischemic stroke who are going to undergo thrombectomy in the

sites participating in the trial will be considered for study enrollment. The principal investigator of each site takes the primary responsibility for patient enrollment. As it is necessary to enroll stroke patients before receiving thrombectomy, it is expected that successful enrollment will require the active participation of relevant physicians at each site. The following factors are expected to limit the enrollment speed:

(1) The absolute number of thrombectomy cases performed.

(2) Compliance with inclusion and exclusion criteria.

(3) Informed consent obtained and baseline evaluation.

4. Inclusion and exclusion criteria

To meet the enrollment criteria of this study, patients must meet the local routine criteria for thrombectomy, and the attending physician should consider the clinical uncertainty of each patient and weigh the possible benefits and risks of tirofiban treatment. In addition to routine site selection for thrombectomy, patients must meet the following study criteria:

4.1 Inclusion criteria

[1] Age 18-85 years (inclusive);

[2] Acute ischemic stroke within 24 hours of onset, and NIHSS >5.

[3] Acute large vessel occlusion, such as internal carotid artery, M1 or M2 segment of middle cerebral artery.

[4] Multimodal CT/MRI imaging completed before thrombectomy, demonstrating penumbra>10ml, infarct core volume <70ml, and mismatch rate >1.2;

[5] Planned to receive endovascular treatment

[6] Informed consent was obtained.

4.2 Exclusion criteria

[1] History of atrial fibrillation or atrial flutter, or 12-lead ECG before randomization and after admission showing atrial flutter or atrial fibrillation.

[2] Treated with intravenous thrombolysis for this stroke or currently taking new oral anticoagulant drugs, warfarin, ticagrelor, and other drugs that may increase the risk of bleeding.

[3] Pre-stroke disability (pre-stroke mRS score > 2).

[4] Severe comorbidity (such as severe cardiopulmonary dysfunction, or the terminal stage of malignant tumors with expected survival less than 90 days).

[5] CT shows hypodensity lesions in more than 1 / 3 of the territory of the middle cerebral artery.

[6] Allergy to tirofiban or having any contraindications to the use of tirofiban (patients with active internal bleeding, history of intracranial hemorrhage, intracranial tumors, arteriovenous malformations, and intracranial aneurysms, or patients resulting in thrombocytopenia from the use of tirofiban previously).

[7] Contraindications to the contrast agents used in multimodal CT/MRI examination (such as allergy to the contrast agents, etc.).

[8] Pregnant or breastfeeding women.

[9] Patients currently participating in other clinical trials.

[10] Other conditions determined by the investigator are not suitable for inclusion in this study.

5. Ethical issues

This study follows the principles stated in the Helsinki Declaration of the World Medical Association.

5.1 Institutional ethics committee approval

Each participating site must obtain written approval(s) from their Institutional Research Ethics Committee (e.g. Institutional Review Board [IRB]) before patient recruitment can commence. Any protocol amendments, serious adverse events (SAEs) reports and routine reporting to the IRB will be the responsibility of the Principal Investigator (PI) at each participating center.

5.2 Informed consent

Most patients with acute large artery ischemic stroke require emergency treatment, including thrombectomy. However, the acute characteristics of this disease mean that patients may not be able understand the study and provide informed consent before emergency treatment. The optional informed consent steps in this study are detailed below, and any local ethics committee requirements should be followed.

5.2.1 Patient informed consent

Whenever possible, written informed consent should be obtained. Clinicians familiar with the study protocol should explain the study to patients and obtain consent to participate in this study.

5.2.2 Surrogate informed consent

If the patient is not fully competent for the consent, for example, because of a reduced level of consciousness, the patient's "surrogate" will be approached to provide informed consent on behalf of the patient.

5.2.3 Withdrawal of consent

The information forms provided to patients and / or their surrogates clearly show that patients can withdraw from the study at any time without explanation, and patients will not be discriminated against or impaired. This situation should be recorded in the patient file. If the patient's withdrawal of consent is only to discontinue tirofiban

medication, further data can be collected and recorded.

5.2.4 Confidentiality and privacy

Patients' privacy should be respected in the conduct of the study. The submitted data will be de-identified to maintain the confidentiality of participants. However, where necessary in the process of monitoring data quality and adherence to the study protocol, the monitors may review medical records at participating hospitals.

6 Randomization

6.1 Randomization procedure

Patients who meet the inclusion criteria are assigned 1:1 to the study group and control group according to the permuted-block randomization method to ensure the balance between and within the groups. Randomization is stratified by participating sites.

Procedures for patients with incorrect inclusion or randomization

If a patient who does not meet the inclusion/exclusion criteria is enrolled in this study, the following procedures should be followed:

1) The investigator or monitors shall immediately notify the physician of the study team to ensure the safety of patients as the primary focus.

2) If continuing the study treatment may endanger the safety of patients, the study treatment must be stopped. After discussion between the study team physician and the investigator, the decision will be made whether to discontinue the study drug. The reasons for discontinuing study treatment must be clearly documented. Patients should be followed up according to the specified study process, including the follow-up of endpoint events until the end of the study, in accordance with the intention-to-treat principle.

3) If continuation of the study is considered not to pose a risk to the patient, nor affect

the treatment of the disease, the reasons for continuing the study should be clearly recorded. Patients should continue to receive follow-up according to the planned study process.

6.2 Blinded follow-up

The follow-up images are uploaded to the local server by each center or the leading center, and the leading center will arrange analysis by trained analysts blinded to treatment group information, including two analysts specialized in neuro-intervention for vascular recanalization, and two analysts mainly engaged in neuroimaging for bleeding analysis.

The clinical evaluation on 90-day follow-up will be performed by a trained professional in the leading center who has no access to the treatment group information of patients.

6.3 Blinded review

After the blinded review and confirmation of the accuracy of the established database, the principal investigator and statistical analyst will lock the data. The locked data is not subject to any change.

7 Interventions

7.1 Study drug

7.1.1 Tirofiban drug selection

Drug name: Tirofiban hydrochloride sodium chloride injection

Drug specifications: 100ml: tirofiban hydrochloride 5mg

7.1.2 Drug label:

Patient number:	

Patient initials:	
Date of use:	
Product No.:	

7.2 Drug dosage and regimen

The study group: intravenous tirofiban combined with thrombectomy. Tirofiban intravenous therapy is initiated within half an hour after randomization and before femoral artery puncture. Tirofiban is used as follows: tirofiban 10 μg/kg, intravenous bolus injection within 3 minutes, and 0.1 μg/kg/min intravenous infusion for 24 hours. The study group is required to maintain tirofiban medication for at least 24 hours according to the protocol. If intracranial hemorrhage is found in follow-up head CT or other serious adverse events (such as gastrointestinal or urinary tract hemorrhage) that may be related to tirofiban within 24 hours, the investigator may consider stopping the medication. The site investigators decide on the subsequent antiplatelet treatment plan based on the patient's condition after 24 hours of intravenous tirofiban infusion.

The control group: treated with routine thrombectomy. The control group is not allowed to use intravenous or intraarterial antiplatelet drugs before the first thrombectomy attempt. Other thrombectomy procedures are carried out in accordance with local guidelines. If there is obvious vascular stenosis or re-occlusion after thrombectomy, the operator can use other rescue measures according to the circumstances, including intra-arterial tirofiban treatment. The rescue treatment does not include an intravenous loading dose of tirofiban.

7.3 Drug management

Drug management should abide by instructions on the drug label and the standard operating procedures for each hospital pharmacy management.

7.4 Procedural method

It is recommended that the time from randomization to femoral artery puncture should be ≤ 90 minutes. During the procedure, all participants are required to undergo a first

thrombectomy attempt after cerebral angiography evaluation. After the first pass thrombectomy, the operator can determine the remaining procedural plan according to the procedural situation. The operator can use other rescue measures at his/her discretion, including balloon dilatation and/or stenting. The control group could use arterial tirofiban rescue treatment after the first pass of thrombectomy, while intra-arterial tirofiban treatment is not recommended for the study group.

Heparinization during the procedure is prohibited.

7.5 Prohibited drugs in the study

Avoid using other intravenous drugs that inhibit platelet aggregation within 24 hours after enrollment, such as ginkgo diterpene lactone injection, ginkgo biloba lipid injection, ginkgo biloba “Damo” injection, Ginkgo biloba extract injection, ozagrel sodium injection, Breviscapine injection, Kudiezi Injection, etc.

8. Withdrawal and exclusion criteria

8.1 Withdrawal decided by the investigator

The investigator can decide to withdraw a patient from the study when the selected participant is not suitable for further study, such as:

(1) Participants with anaphylaxis or serious adverse events who should stop the study at the investigator’s discretion.

(2) Deterioration of the participant's symptoms after medication. To protect the participant, the participant will be withdrawn from the study and accept other effective treatments.

(3) Participant developing new comorbidities, complications, or special physiological changes, which affect the efficacy and safety evaluation.

(4) If, due to poor compliance, the dosing of drugs is less than 70% or more than 130% of the prescribed dose.

(5) Other reasons indicating it is inappropriate to continue the study.

8.2 Withdrawal decided by the participant

According to the informed consent, participants have the right to withdraw from the study at any time. A participant who is lost to follow-up precluding further medication or testing, will also be considered a "withdrawal" (or "drop-off") although the subject has not explicitly expressed a desire to withdraw from the study.

Patients are free to withdraw from the study at any time (e.g., permanent withdrawal of study drugs and withdrawal from study evaluation), without affecting their further treatment. The withdrawal of informed consent from this study must be confirmed by the investigator and recorded on the patient file and informed consent form. If possible, patients and investigators should re-sign and date the informed consent form. These patients will be asked about the reasons for withdrawal and whether there are adverse events. The reason for permanent discontinuation of the study drug use and the date of the last dose of study drug must be recorded in the patient file.

Patients who permanently discontinued the study drug should receive routine treatment, and if applicable, participate in follow-up visits for the study. If the patient refuses any additional study follow-up and formally withdraws their consent (including whether he/she agrees to continued use of collected clinical data without identity information), the leading center shall be informed at the same time. If the patient and family members refuse to provide any clinical data for the study, the leading center will contact the database to delete all the data of the patient after being notified.

At the end of the originally planned follow-up period of all patients (90 days after randomization), the survival status of patients who withdrew their informed consent will be collected from public sources within the scope allowed by local regulations after their withdrawal of informed consent.

To ensure the validity of the study data, it is important to collect as much data as

possible during the study, especially the survival status (death or survival). Therefore, the investigator should try to collect the survival status information of all patients who withdrew informed consent through public source data at the end of treatment visit and the study closure visit. Participants who withdraw cannot be replaced.

8.3 Criteria of exclusion from data analysis

Participants who should not be enrolled but have been enrolled, or who have completed the study but have violated some provisions of the study protocol, should be excluded from data analysis according to the investigator, including:

(1) Those who have not used the prescribed medication.

(2) Those without any test results after medication.

(3) Those in whom, due to the use of forbidden drugs, the efficiency and safety cannot be evaluated.

At the blinded review meeting, the principal investigator and statistician will decide whether to eliminate cases and what data set to enter. The reasons for the exclusion should be explained, and their medical notes should be retained for future reference.

9. Study outcomes:

Primary outcome:

A composite primary outcome is defined as first-pass recanalization of targeted vessels without increasing the risk of symptomatic intracranial hemorrhage. "First-pass recanalization" is defined as (1) single pass/use of the device, (2) basic or complete revascularization of the targeted vessels (mTICI ≥ 2b) after the first pass, and before other endovascular treatments (stent, balloon dilation, or platelet therapy). If the first DSA angiography before the first attempt of thrombectomy demonstrates mTICI grade ≥ 2b, this will also be considered achieving “first-pass recanalization”. Symptomatic intracranial hemorrhage was defined according to SITS-MOST criteria.

Secondary outcomes:

- The recanalization rate of targeted vessels after thrombectomy.
- The recanalization rate of targeted vessels at the end of endovascular treatment.
- The recanalization rate of targeted vessels at 24-72 hours.
- Symptomatic intracranial hemorrhage within 72 hours.
- 90-day functional outcomes (dichotomized 90-day mRS, ordinal scale of 90-day mRS).
- Death at 90 days.
- Number of thrombectomy passes required.

10. Data collection and follow-up

The study sites are required to collect data on patients at baseline (before randomization); treatment period (day 0; the day of randomization); 24-72 hours after randomization; day 7 (or at discharge if discharged earlier) and 90 days (± 7 days) after randomization. All SAEs including death within the 3-month follow-up period are recorded. The assessments at 90-days are to be undertaken by a well-trained investigator, who is blind to treatment allocation, either face to face or by telephone. Source documents, such as patient interview records or questionnaire responses, can be directly entered into the database. Paper case report forms can also be filled in and retained as source documents. Brain imaging will be collected at baseline, during the treatment period, and 24-72 hours (± 8 hours). If clinically indicated, patients may receive further brain imaging scans at a later stage. Brain imaging data are collected by each site. The leading site assists each site in uploading image data. After uploading, the leading site is responsible for image analysis.

90-day follow-up is required for all participants in this study. If they die before 90 days, they will be followed up until death. Patients who do not comply with the protocol

and/or terminate the assigned treatment regimen also need to be followed up for 90 days, as their data will be required for the intention-to-treat analysis. The study flow chart lists the types of data and the schedule of evaluation. The leading site will provide the paper version of the case report form and the instruction manual for reference, as well as the guidelines of the case report form and the definition of terms.

10.1 Patient Contact Details log

Each center will keep a record of the contact details and information of next-of-kin for all patients recruited into the study. This will be kept with the patient file in the participating center in a locked filing cabinet in accordance with local privacy and confidentiality policies. The patient contact detail log will also be used to record any issues in the process of informed consent, work done during follow-up, and protocol violations. This will be used by the local research coordinator and PI to record the management of the informed consent process, and follow-up arrangements, and respond to queries from the leading center.

10.2 Randomization assessment

All patients with acute ischemic stroke due to large artery occlusion will be assessed for eligibility for the study using a checklist of the criteria described previously. The enrollment and randomization log should be kept in a locked filing cabinet within each participating center.

The log should include:

- Patient demographic information: sex, date of birth, time of onset (or last seen without symptoms), time of admission, NIHSS before randomization, pre-stroke mRS.
- Imaging evaluation before randomization: CTP time, occluded vessel, penumbra volume, core infarct volume, and mismatch ratio.
- Randomization stratification information: site number.

- Inclusion and exclusion criteria checklist.
- Randomization information.

10.3 Baseline data

- Medical history: hypertension, diabetes mellitus, stroke, ischemic heart disea se, hypercholesterolemia, smoking and drinking history, coagulation dysfunct ion or other blood diseases, history of gastrointestinal or urinary tract bleed ing, previous oral drugs (aspirin, clopidogrel, warfarin, antihypertensive dru gs, lipid-lowering drugs, antidiabetic drugs), etc.
- Vital signs: blood pressure, heart rate (HR), weight, height, GCS, and NIHSS at admission.
- Blood test results:
  - Full blood count: red blood cells, white blood cells, neutrophils (count, percentage), hematocrit, hemoglobin, platelets.
  - Serum biochemistry: renal function: creatinine, urea (or urea nitrogen), uric acid; liver function: glutamic oxaloacetic transaminase, glutamic pyruvic transaminase; glucose.
  - Coagulation function: PT, APTT, TT, FIB, INR, D-dimer, fibrin degradation products.
- Brain imaging: brain multimodal CT.

10.4 Treatment information (0-24h)

- Tirofiban administration information, including tirofiban intravenous bolus start time, intravenous bolus dose, intravenous infusion start time, and intravenous infusion end time. Ensure that the patient treatment complies with their randomization.
- Endovascular treatment information, including the mTICI grade of targeted vessels after the first angiography, mTICI grade of targeted vessels after the first-

pass thrombectomy, thrombectomy devices (second-generation, third-generation, aspiration or other), degree of stenosis after thrombectomy, number of thrombectomy passes, mTICI grade of targeted vessels after thrombectomy, use or otherwise of balloon dilatation or stent implantation, the final mTICI grade at the end of endovascular thrombectomy, and the final residual stenosis (0-30%; 30-50%; 50-70%, 70-99%). Intraoperative complications, femoral artery puncture time, DSA first angiography time, final recanalization time.

- Brain imaging: DSA collected the endovascular treatment process.
- Concomitant medication: antiplatelet drugs (aspirin, clopidogrel, cilostazol, others); Anticoagulant drugs.

10.5 Follow-up data

10.5.1 24-72 hours after randomization

- GCS and NIHSS scores
- Brain imaging follow-up (brain non-contrast CT and CTA; if brain CTA cannot be acquired, brain MRA is acceptable; if the patient has extracranial occlusion of the internal carotid artery, it is necessary to acquire carotid artery CTA or MRA at the same time), and the AOL grade on the brain CTA or MRA
- Blood test results:
  - Full blood count: red blood cells, white blood cells, neutrophils (count, percentage), hematocrit, hemoglobin, platelet count;
  - Serum biochemistry: renal function: creatinine, urea (or urea nitrogen), uric acid; liver function: glutamic oxaloacetic transaminase, glutamic pyruvic transaminase; glucose.
  - Coagulation function test: PT, APTT, TT, FIB, INR, D-dimer, fibrinogen degradation products.

- Clinical safety events: gastrointestinal bleeding, urinary tract bleeding, thrombocytopenia, coagulation dysfunction.

10.5.2 Discharge assessment form (up to 90-day at the latest)

On the day of discharge or death, confirm and record the contact details of the patient or the caregiver for follow-up evaluation in the future.

The following shall be recorded:

- Discharge time
- GCS, NIHSS, and mRS scores at discharge

10.5.3 90 days (± 7)

- mRS score

These assessments should be conducted by an investigator who was not involved in the clinical management of patients and is blinded to the randomized treatment allocation. At 90-day ± 7 days, all surviving patients will be consulted and evaluated by telephone or face-to-face.

10.5.4 Death

If the patient dies before the planned follow-up time, details of the cause of death of the patient, including the date and time of death, shall be recorded. Copies of autopsy reports, admission records, or death certificates should be kept together with the participant contact form for monitoring by the leading center.

10.6 Discontinuation of assigned treatment and protocol violation

The leading center will provide a form to record the date and details of deviations from the protocol or missing assessments.

10.7 Informed consent

The process of informed consent should be recorded in the patient's medical record and case report form. The type of informed consent obtained should be recorded in the database.

10.8 Serious adverse events

All SAEs shall be recorded in the SAE form and reported to the leading center within the specified time by fax or e-mail. Additional information on events and outcomes may be requested.

VII. Study flow chart

Evaluation	Baseline (screening and enrollment)	Treatment period (0-24 hours)	24-72 hours after randomization	At discharge (up to 90 days at the latest)	Day 90 ± 7 (Final visit)
Informed consent	X				
Demographic characteristics	X				
Symptoms of this stroke	X				
Medical history	X				
Concomitant medication	X	X	X	X	
Critical time[1]	X	X			
Vital signs	X	X			
Laboratory examination	X		X		
Electrocardiogram	X				
Inclusion and exclusion criteria	X				
Randomization	X				
Imaging examination	X		X		
Tirofiban treatment information		X			
Thrombectomy information		X			
mRS	X			X	X
NIHSS	X		X	X	

GCS	X		X	X	
SAEs		X	X	X	X

1.critical time: onset time (or last seen well time) at randomization assessment, time of admission, time of CTP; The start time of tirofiban intravenous bolus, the start time of intravenous infusion, the end time of intravenous infusion, the femoral artery puncture time, the first DSA angiography time, and the final recanalization time of endovascular treatment.

VIII. Serious adverse events

1. Definition:

The mechanisms for reporting and notifying SAE are based on the guidelines adopted by the ICH-GCP. As defined by the WHO International Drug Monitoring Centre (1994), an SAE is any untoward medical occurrence that:

1) Causes death;

2) Is life-threatening (i.e. the patient was at risk of death at the time of the event; it does not refer to an event that might hypothetically have caused death had it been more severe).

3) Needs hospitalization or extension of hospitalization.

4) Causes persistent or significant disability or incapacity.

5) Results in congenital anomaly or birth defect (NB, females in the study population are likely to be post-menopausal).

6) Is a major medical event judged by the PI that is not immediately life-threatening and does not result in death or hospitalization but may jeopardize the patient or may require intervention to prevent one of the other outcomes listed above.

Note: the following situations are not reported as SAEs:

1) Scheduled elective surgery before signing the informed consent for this study;

2) Hospitalization required by routine health examination (such as routine gastrointestinal endoscopy);

3) Hospitalized for neurological rehabilitation only.

Unexpected adverse reactions (UARS) are adverse reactions that do not conform to the drug instructions. Suspected unexpected serious adverse reaction (SUSAR) is an unexpected adverse reaction at any dose, which:

1) Causes death

2) Is life-threatening (i.e. the patient was at risk of death at the time of the event; it does not refer to an event that might hypothetically have caused death had it been more severe)

3) Needs hospitalization or extension of hospitalization

4) Cause persistent or significant disability or incapacity

5) Results in congenital anomaly or birth defect.

2. Record and report on SAEs

An SAE form must be used to record the details of the event, and this will include a full description of the event, classification of the event using the above definitions, the PI's opinion on the causal relationship to the randomized management group, and the timing of the event. All SAEs should be reported to the leading site within 24 hours after the event is recognized. To obtain further information, the principal investigator may need to submit a tracking report to record the results of SAEs. The principal investigator is responsible for reporting SAEs to the institutional ethics committee according to local regulations.

A SUSAR form must be used to record the details of the event, including the full description of the event, the classification of the event according to the above definitions, the judgment of the PI on the causal relationship between the SUSAR and the suspected drug, and the details of the suspected drug. All SUSAR should be reported to the leading site within 24 hours as soon as the event is recognized. The principal investigator may need to provide more details to supplement the initially reported serious adverse events, and the results of SUSAR must also be recorded. The principal investigator is responsible for reporting SUSAR to the IRB or management department according to local regulations or management requirements.

3. Monitoring of SAEs

The leading center will closely monitor all SAEs to understand the relationship between

SAEs and study procedures and protocols, or the clustering of similar events in a specific location. If there are too many special SAEs related to the protocol, the study protocol needs to be revised or the trial needs to be terminated early. In addition, beyond the planned interim analysis meeting, the leading center will submit all SUSAR information to the data safety monitoring board for review.

4. Monitoring of SUSAR

The leading center will closely monitor all SUSAR to understand the relationship between SUSAR and study procedures and protocols, or the occurrence of similar events in a specific trial center. In addition, the leading center will report according to the requirements of the independent ethics committees of the participating centers.

IX. Quality assurance

The study will be conducted in hospitals with acute stroke units and experience in thrombectomy. The monitor will check the data of each site and supervise the implementation of the trial. The first site monitoring visit will be conducted after the first few patients are randomized by a center, and then at least twice a year according to the number of selected patients. Through monitoring, we can ensure that investigators can strictly comply with the protocol and GCP requirements, can ensure the authenticity and reliability of the data, and ensure that all conclusions in the clinical trial are derived from the original data.

1. Preservation and supervision of original data/documents

The original documents are the basis for the true existence of the subjects and the credibility of the collected data. The original documents should be kept in each center.

The data on the case report form is from the original document and should be consistent with the source document. If there is any discrepancy, the reason should be explained.

All other data (i.e. written or electronic text records without data) can be directly

recorded on the case report form and recognized as original data.

The investigator must allow the initiator to conduct supervision, inspection, review by the ethics committee, and inspection by relevant management departments, and allow the above personnel to access all relevant original materials/documents.

The case report form and all original materials, including laboratory and other medical examination results, must be ready for review by the clinical monitor designated by the initiator at any time. The clinical monitor should check all case report forms and informed consent forms to confirm the accuracy of the data in the documents.

2. Preparation before the study

Before the first participant is enrolled in the study, the representative of the study initiator should visit the center and should:

1. determine whether the facilities of the center are sufficient;

2. determine whether there are suitable enrolled patients at the study site;

3. discuss with the investigator/director of the center (and other related personnel) their responsibilities of the protocol and the study initiator or its representative, and record them in the clinical study agreement of the study initiator and the investigator/director of the center.

3. Site staff training

Before the first subject is enrolled for the study, the leading site will conduct standardized training and assessment for the principal investigators and all investigators in all sites, and the study in all sites can be started only after passing the assessment. The principal investigator shall ensure that all investigators receive study-related training and make training records.

The principal investigator will keep a training record of all related personnel (doctors, nurses, and others).

4. Study monitoring

During the study, the initiator will contact the site regularly, including:

1. Visit the site to provide information and support for investigators

2. Confirm that the facilities continue to meet the requirements

3. Confirm that all related personnel comply with the study protocol, the data records on the case report form are timely and accurate, and the accountability of study drugs is correct.

4. Conduct source data verification (SDV) (compare the data on the case report form to the subjects' records in the hospital and other records related to the study), including the review of the participant's informed consent. It is necessary to review all original records of each patient (such as inpatient medical records).

If the investigator or other staff of the site need to know the information and advice about the implementation of the study, they can contact the initiator of the study during the follow-up period.

When the study is completed, the monitor should ensure that each center has a plan for the long-term (15 years) preservation of relevant data and source documents.

5. Supervision and inspection by government regulators

In addition, the study may also be audited by a third-party organization and by inspectors dispatched by the government administration. During and after the study, all sites must ensure that medical record report forms, original documents, and other study documents can be reviewed during the inspection auditing.

6. Source data

The study sites will retain all original records and source data.

7. Archiving of study documents

The investigator should abide by the principles listed in the clinical study agreement.

8. Study progress

If the study procedures are not carried out according to the trial protocol, or the recruitment period is too long, the study of the site may be terminated, and the initiator of the study will also terminate the study in advance due to the safety issues in this study or other tirofiban injection studies.

X. Data management

Data entry will be completed by the participating sites through the web-based data management system with password protection commissioned by the leading site. Paper case report forms will be provided to centers willing to use them for preliminary data collection.

XI. Statistics

1. statistical considerations

The intention to treat (ITT) principle will be applied in analyses as the full analysis set. Full analysis set: according to the basic principle of (ITT) analysis, all randomized subjects with more than one medication record and effectiveness evaluation will be included in the full analysis set. In the analysis of the full analysis set, the carry forward estimation method of the latest observation can be used for the estimation of missing values. The full analysis set is the main effectiveness evaluation population of this study.

In addition, this study will also establish a per-protocol (PP) set: including all subjects who complete the treatment specified in the protocol or do not have major protocol violations. The definition of major protocol violation will be finalized at the time of data review, which may generally include the following cases (but not limited to these

cases): serious violation of the inclusion criteria, treatment that seriously interferes with the efficacy evaluation after enrollment, serious violation of the medication dosing scheme, loss of the primary efficacy evaluation, etc.

2. General principles

A superiority test will be conducted in this study. Two-sided $\alpha=0.05$, and $p<0.05$ is considered as a statistical significance threshold. All statistical analyses will be processed with Stata 13.0 or higher statistical software.

3. Analysis

The statistical analysis includes (1) the number of cases completed at each site and the withdrawal of cases; (2) analysis of demographics and baseline characteristics of patients in each group at the time of enrollment, and the comparability between the study group and the control group; (3) efficacy evaluation including the determination of efficacy endpoints and the efficacy comparison of each group; (4) safety evaluation including the comparison of laboratory results and clinical adverse reactions in each group.

4. Primary outcome and sample size estimation

This study is a 1:1 randomized controlled trial. The study group is the tirofiban combined with thrombectomy group, and the control group is the routine thrombectomy group. The primary outcome of the study is a composite outcome: the probability of first-pass recanalization of targeted vessels without increasing the risk of symptomatic intracranial hemorrhage. The Chi-square test is used to compare the primary endpoint between groups.

According to the retrospective analysis of the International Stroke Perfusion Imaging registry (INSPIRE https://taste.newcastle.edu.au/key=inspire) participated in by the research team, the proportion of patients who achieved first-pass recanalization without

symptomatic hemorrhage using intravenous tirofiban before thrombectomy was 71% (94/132), while the rate of patients who achieved first-pass recanalization without symptomatic hemorrhage in direct thrombectomy was 48% (55/115).

The conservative estimate for this project is that the rate of primary outcome will be 70% in the study group and 50% in the control group. With two-sided $\alpha=0.05$, power=80%, assuming a 7% dropout rate, the final sample size is 100 subjects in the study group and 100 subjects in the control group, or 200 subjects in total, which was calculated using Stata 13.0 software.

XII. Publications and reports

Publication of the main reports from the study will be in the name of the principal investigator. Full editorial control will reside with a Writing Committee approved by the Steering Committee (SC). Investigators have the right to publish or present the results of the study. However, as this is a multicenter study, investigators must agree not to publish or publicly present any interim results of the study without the prior written permission of the SC. Investigators must further agree to provide the SC at least 30 days prior notice of any submission for publication or presentation for review, copies of abstract or manuscripts (including without limitation, text and PowerPoint presentation slides and any other texts of translations or media presentations) that reports any study results.

The SC has the right to review and evaluate published articles, abstracts, slides and manuscripts from the perspective of accuracy of information, protection of personal rights and ensuring that any output is properly weighed and comply with relevant regulations.

If the parties disagree on the suitability and / or confidentiality of data analysis and presentation, the investigators agree to meet with the members of the SC at the clinical center or an agreed place before submission for publication to discuss and attempt to resolve any such issues or differences in good faith.

The writing committee will be composed of committee members, statisticians, and investigators, and the honor belongs to the cooperative investigators and other investigators.

Authorship of published articles must comply with the following guidelines of the international medical journal editorial board for authors.

1 An author must make substantial contributions to the conception and design of the trial, the acquisition or analysis of the data, and the interpretation of the results.

2 An author must personally draft and publish the article or make contributions to the important revision of the original manuscript (data analysis, interpretation or other important knowledge content) during the manuscript review process and obtain the approval of other authors.

3 Before submitting the article to the journal for publication, the author must provide the approval of the final draft version of the original manuscript.

According to the acknowledgement guidelines of the international medical journal editorial board, if the journal allows, all contributors who do not meet the above three criteria for authorship will be listed in the acknowledgments in the publication.

XIII. Organization

This study is initiated and executed by investigators and managed by Shanghai East Hospital as the leading site.

Participating sites

Neurology ward / Department of Neuroscience / acute stroke unit / Neurosurgery

Responsibilities: overall manage the study of respective hospitals and ensure the compliance of the study plan; Recruiting and training study nurses; Introduce the protocol to colleagues, enroll patients, collect data, solve data queries, contact the local hospital ethics committee / institutional review board according to the local ethical guidelines and relevant reporting requirements, and report adverse events to the local

hospital ethics committee / institutional review board and the leading center according to the protocol.

Data Safety Monitoring Board

In this study, a data safety monitoring board will be established to regularly review the unblinded data in the study follow-up, and the dropout rate and event rate will be monitored. The detailed statistical analysis plan will be completed and archived before the enrollment of 50 patients, which will provide the details of stopping principles.

XIV. Funding

This study receives funding from Shanghai east hospital and the stroke and dementia special committee of the Shanghai Science and Technology Development Foundation.

XV. Timeline

Site ethics committee submission in January 2021

Initiating meetings, screening, and enrollment in March 2021

End of follow-up December 2023

Statistical analysis, publication in June 2024

XVI. References

1. Collaborators GBDS. Global, regional, and national burden of stroke, 1990-2016: A systematic analysis for the global burden of disease study 2016. *Lancet Neurol*. 2019;18:439-458
2. Collaborators GBDLRoS, Feigin VL, Nguyen G, Cercy K, Johnson CO, Alam T, et al. Global, regional, and country-specific lifetime risks of stroke, 1990 and 2016. *N Engl J Med*. 2018;379:2429-2437
3. Kelly-Hayes M, Beiser A, Kase CS, Scaramucci A, D'Agostino RB, Wolf PA. The influence of gender and age on disability following ischemic stroke: The framingham study. *J Stroke Cerebrovasc Dis*. 2003;12:119-126
4. Wu S, Wu B, Liu M, Chen Z, Wang W, Anderson CS, et al. Stroke in china: Advances and challenges in epidemiology, prevention, and management. *Lancet Neurol*. 2019;18:394-405
5. Powers WJ. Acute ischemic stroke. *N Engl J Med*. 2020;383:252-260
6. Manning NW, Campbell BC, Oxley TJ, Chapot R. Acute ischemic stroke: Time, penumbra, and reperfusion. *Stroke*. 2014;45:640-644
7. Campbell BCV, De Silva DA, Macleod MR, Coutts SB, Schwamm LH, Davis SM, et al. Ischaemic stroke. *Nat Rev Dis Primers*. 2019;5:70 doi: 10.1038/s41572-019-0118-8.
8. Phipps MS, Cronin CA. Management of acute ischemic stroke. *BMJ*. 2020;368:l6983 doi: 10.1136/bmj.l6983.
9. Emberson J, Lees KR, Lyden P, Blackwell L, Albers G, Bluhmki E, et al. Effect of treatment delay, age, and stroke severity on the effects of intravenous thrombolysis with alteplase for acute ischaemic stroke: A meta-analysis of individual patient data from randomised trials. *Lancet*. 2014;384:1929-1935
10. Goyal M, Demchuk AM, Menon BK, Eesa M, Rempel JL, Thornton J, et al. Randomized assessment of rapid endovascular treatment of ischemic stroke. *New Engl J Med*. 2015;372:1019-1030
11. Jovin TG, Chamorro A, Cobo E, de Miquel MA, Molina CA, Rovira A, et al. Thrombectomy within 8 hours after symptom onset in ischemic stroke. *New Engl J Med*. 2015;372:2296-2306
12. Nogueira RG, Jadhav AP, Haussen DC, Bonafe A, Budzik RF, Bhuva P, et al. Thrombectomy 6 to 24 hours after stroke with a mismatch between deficit and infarct. *New Engl J Med*. 2018;378:11-21
13. Albers GW, Marks MP, Kemp S, Christensen S, Tsai JP, Ortega-Gutierrez S, et al. Thrombectomy for stroke at 6 to 16 hours with selection by perfusion imaging. *New Engl J Med*. 2018;378:708-718

14. Kellert L, Hametner C, Rohde S, Bendszus M, Hacke W, Ringleb P, et al. Endovascular stroke therapy tirofiban is associated with risk of fatal intracerebral hemorrhage and poor outcome. *Stroke*. 2013;44:1453-5
15. Teng D, Pannell JS, Rennert RC, Li J, Li YS, Wong VW, et al. Endothelial trauma from mechanical thrombectomy in acute stroke: In vitro live-cell platform with animal validation. *Stroke*. 2015;46:1099-1106
16. Adeoye O, Sucharew H, Khoury J, Vagal A, Schmit PA, Ewing I, et al. Combined approach to lysis utilizing eptifibatide and recombinant tissue-type plasminogen activator in acute ischemic stroke-full dose regimen stroke trial. *Stroke*. 2015;46:2529-2533
17. Saver JL, Goyal M, Bonafe A, Diener HC, Levy EI, Pereira VM, et al. Stent-retriever thrombectomy after intravenous t-pa vs. T-pa alone in stroke. *N Engl J Med*. 2015;372:2285-2295
18. Campbell BCV, Mitchell PJ, Kleinig TJ, Dewey HM, Churilov L, Yassi N, et al. Endovascular therapy for ischemic stroke with perfusion-imaging selection. *New Engl J Med*. 2015;372:1009-1018
19. Soares BP, Tong E, Hom J, Cheng SC, Bredno J, Boussel L, et al. Reperfusion is a more accurate predictor of follow-up infarct volume than recanalization: A proof of concept using ct in acute ischemic stroke patients. *Stroke*. 2010;41:E383-E383
20. Falk E. Plaque rupture with severe pre-existing stenosis precipitating coronary thrombosis. Characteristics of coronary atherosclerotic plaques underlying fatal occlusive thrombi. *Br Heart J*. 1983;50:127-134
21. Ruggeri ZM. Structure and function of von willebrand factor. *Thromb Haemost*. 1999;82:576-584
22. Ruggeri ZM. Platelet adhesion under flow. *Microcirculation*. 2009;16:58-83
23. McFadyen JD, Jackson SP. Differentiating haemostasis from thrombosis for therapeutic benefit. *Thromb Haemost*. 2013;110:859-867
24. Jin J, Daniel JL, Kunapuli SP. Molecular basis for adp-induced platelet activation. Ii. The p2y1 receptor mediates adp-induced intracellular calcium mobilization and shape change in platelets. *J Biol Chem*. 1998;273:2030-2034
25. Matsui Y, Amano H, Ito Y, Eshima K, Suzuki T, Ogawa F, et al. Thromboxane a2 receptor signaling facilitates tumor colonization through p-selectin-mediated interaction of tumor cells with platelets and endothelial cells. *Cancer Sci*. 2012;103:700-707
26. Huang JS, Ramamurthy SK, Lin X, Le Breton GC. Cell signalling through thromboxane a2 receptors. *Cellular signalling*. 2004;16 (5):521-33. doi: 10.1016/j.cellsig.2003.10.008.
27. Coughlin SR. How the protease thrombin talks to cells. *P Natl Acad Sci USA*. 1999;96:11023-11027
28. Bennett JS. Structure and function of the platelet integrin alphaiibbeta3. *The Journal of clinical investigation*. 2005;115 (12):3363-9.doi: 10.1172/JCI26989.

29. Nieswandt B, Varga-Szabo D, Elvers M. Integrins in platelet activation. *J Thromb Haemost*. 2009;7:206-209
30. Furie B, Furie BC. Mechanisms of disease: Mechanisms of thrombus formation. *New Engl J Med*. 2008;359:938-949
31. McFadyen JD, Schaff M, Peter K. Current and future antiplatelet therapies: Emphasis on preserving haemostasis. *Nat Rev Cardiol*. 2018;15:181-191
32. Roth GJ, Stanford N, Majerus PW. Acetylation of prostaglandin synthase by aspirin. *Proc Natl Acad Sci U S A*. 1975;72:3073-3076
33. Loll PJ, Picot D, Garavito RM. The structural basis of aspirin activity inferred from the crystal structure of inactivated prostaglandin h2 synthase. *Nat Struct Biol*. 1995;2:637-643
34. Wallentin L. P2Y(12) inhibitors: Differences in properties and mechanisms of action and potential consequences for clinical use. *Eur Heart J*. 2009;30:1964-1977
35. Yusuf S, Zhao F, Mehta SR, Chrolavicius S, Tognoni G, Fox KK. Clopidogrel in Unstable Angina to Prevent Recurrent Events Trial Investigators. Effects of clopidogrel in addition to aspirin in patients with acute coronary syndromes without st-segment elevation. *New Engl J Med*. 2001;345 (7):494-502. doi: 10.1056/NEJMoa010746.
36. Kalz J, ten Cate H, Spronk HMH. Thrombin generation and atherosclerosis. *J Thromb Thrombolys*. 2014;37:45-55
37. Tricoci P, Huang Z, Held C, Moliterno DJ, Armstrong PW, Van de Werf F, et al. Thrombin-receptor antagonist vorapaxar in acute coronary syndromes. *New Engl J Med*. 2012;366:20-33
38. Muñiz-Lozano A, Rollini F, Franchi F, Angiolillo DJ. Update on platelet glycoprotein iib/iiia inhibitors: Recommendations for clinical practice. *Therapeutic Advances in Cardiovascular Disease*. 2013;7(4):197-213. doi: 10.1177/1753944713487781.
39. Zhu JZ, Hu J. Clinical application Progress in non peptide gp Ⅱ b/Ⅲ a receptor antagonists. *The Medical Forum*. 2014;18:2557-2559
40. Bazzino O, Aylward P, Hains A, Slany J, Steinbach K, Van de Werf F, et al. A comparison of aspirin plus tirofiban with aspirin plus heparin for unstable angina. *New Engl J Med*. 1998;338:1498-1505
41. Bazzino O, Barrero C, Garre L, Sosa A, Aylward P, Slany J, et al. Inhibition of the platelet glycoprotein iib/iiia receptor with tirofiban in unstable angina and non-q-wave myocardial infarction. *New Engl J Med*. 1998;338:1488-1497
42. Zhang Y, Gao CY, Zhu ZY, Liu HY, Wang XP, Yang HH, et al. The efficacy and safety of immediate use of tirofiban after successful interventional treatment in patients with medium to high risk non ST segment elevation acute coronary syndrome. *Chinese Journal of Cardiology.* 2013;41:731-735
43. Yang L, Yang XC, Wang LF, Ge YG, Wang SH, Li WM, et al. The effect of early application of tirofiban on the efficacy of emergency interventional therapy in patients with acute ST segment elevation myocardial infarction. *Chinese Journal of Cardiology*. 2006;983-986

44. *Roffi M, Chew DP, Mukherjee D, Bhatt DL, White JA, Moliterno DJ,* et al. Platelet glycoprotein iib/iiia inhibition in acute coronary syndromes. Gradient of benefit related to the revascularization strategy. *Eur Heart J.2002; 23(18):1441-8. doi: 10.1053/euhj.2002.3160.*
45. Luca GD, Navarese E, Marino P. Risk profile and benefits from Gp IIb-IIIa inhibitors among patients with st-segment elevation myocardial infarction treated with primary angioplasty: A meta-regression analysis of randomized trials. *Eur Heart J*. 2009;30(22):2705-13. doi: 10.1093/eurheartj/ehp118.
46. Wang HL, Xing SY, Dong PS, Han YH, Zhu JH, Lai LH, et al. Safety and efficacy of intracoronary tirofiban administration in patients with serious thrombus burden and st-elevation myocardial infarction undergoing percutaneous coronary intervention. *European review for medical and pharmacological sciences*. 2014;18 (23):3690-5.
47. Berg JMt, Hof AWJvt, Dill T, Heestermans T, Werkum JWv, Mosterd A, et al. Effect of early, pre-hospital initiation of high bolus dose tirofiban in patients with st-segment elevation myocardial infarction on short- and long-term clinical outcome. *Journal of the American College of Cardiology*. 2010;55(22):2446-55.
48. Akpek M, Sahin O, Sarli B, Baktir AO, Saglam H, Urkmez S, et al. Acute effects of intracoronary tirofiban on no-reflow phenomena in patients with st-segment elevated myocardial infarction undergoing primary percutaneous coronary intervention. *Angiology*. 2015;66
49. van't Hof AWJ, ten Berg J, Heestermans T, Dill T, Funck RC, van Werkum W, et al. Prehospital initiation of tirofiban in patients with st-elevation myocardial infarction undergoing primary angioplasty (on-time 2): A multicentre, double-blind, randomised controlled trial. *Lancet*. 2008;372:537-546
50. Yang M, Huo X, Gao F, Wang A, Ma N, Shi H, et al. Low-dose rescue tirofiban in mechanical thrombectomy for acute cerebral large-artery occlusion. *Eur J Neurol*. 2020;27:1056-1061
51. Yang J, Wu Y, Gao X, Bivard A, Levi CR, Parsons MW, et al. Intraarterial versus intravenous tirofiban as an adjunct to endovascular thrombectomy for acute ischemic stroke. *Stroke*. 2020;51:2925-2933
52. Huo X, Yang M, Ma N, Gao F, Mo D, Li X, et al. Safety and efficacy of tirofiban during mechanical thrombectomy for stroke patients with preceding intravenous thrombolysis. *Clin Interv Aging*. 2020;15:1241-1248
53. Zhang Y, Zhang QQ, Fu C, Wang L, Zhang GQ, Cao PW, et al. Clinical efficacy of tirofiban combined with a solitaire stent in treating acute ischemic stroke. *Braz J Med Biol Res*. 2019;52:e8396
54. Zhang S, Hao Y, Tian X, Zi W, Wang H, Yang D, et al. Safety of intra-arterial tirofiban administration in ischemic stroke patients after unsuccessful mechanical thrombectomy. *J Vasc Interv Radiol*. 2019;30:141-147 e141
55. Yi HJ, Sung JH, Lee DH. Safety and efficacy of intra-arterial tirofiban injection during mechanical thrombectomy for large artery occlusion. *Curr Neurovasc Res*. 2019;16:416-424

56. Sun C, Li X, Zhao Z, Chen X, Huang C, Li X, et al. Safety and efficacy of tirofiban combined with mechanical thrombectomy depend on ischemic stroke etiology. *Front Neurol*. 2019;10:1100
57. Quan T, Hou H, Xue W, Yu G, Ma H, Sun J, et al. Endovascular treatment of acute intracranial vertebrobasilar artery occlusion: A multicenter retrospective observational study. *Neuroradiology*. 2019;61:1477-1484
58. Pan X, Zheng D, Zheng Y, Chan PWL, Lin Y, Zou J, et al. Safety and efficacy of tirofiban combined with endovascular treatment in acute ischaemic stroke. *Eur J Neurol*. 2019;26:1105-1110
59. Neuberger U, Seker F, Schonenberger S, Nagel S, Ringleb PA, Bendszus M, et al. Prediction of intracranial hemorrhages after mechanical thrombectomy of basilar artery occlusion. *J Neurointerv Surg*. 2019;11:1181-1186
60. Luo Y, Yang Y, Xie Y, Yuan Z, Li X, Li J. Therapeutic effect of pre-operative tirofiban on patients with acute ischemic stroke with mechanical thrombectomy within 6-24 hours. *Interv Neuroradiol*. 2019;25:705-709
61. Liu J, Shi Q, Sun Y, He J, Yang B, Zhang C, et al. Efficacy of tirofiban administered at different time points after intravenous thrombolytic therapy with alteplase in patients with acute ischemic stroke. *J Stroke Cerebrovasc Dis*. 2019;28:1126-1132
62. Gruber P, Hlavica M, Berberat J, Victor Ineichen B, Diepers M, Nedeltchev K, et al. Acute administration of tirofiban versus aspirin in emergent carotid artery stenting. *Interv Neuroradiol*. 2019;25:219-224
63. Yu T, Lin Y, Jin A, Zhang P, Zhou X, Fang M, et al. Safety and efficiency of low dose intra-arterial tirofiban in mechanical thrombectomy during acute ischemic stroke. *Curr Neurovasc Res*. 2018;15:145-150
64. Wu Y, Yin C, Yang J, Jiang L, Parsons MW, Lin L. Endovascular thrombectomy. *Stroke*. 2018;49:2783-2785
65. Kang DH, Yoon W, Kim SK, Baek BH, Lee YY, Kim YW, et al. Endovascular treatment for emergent large vessel occlusion due to severe intracranial atherosclerotic stenosis. *J Neurosurg*. 2018:1-8
66. Zhao W, Che R, Shang S, Wu C, Li C, Wu L, et al. Low-dose tirofiban improves functional outcome in acute ischemic stroke patients treated with endovascular thrombectomy. *Stroke*. 2017;48:3289-3294
67. Zhao H, Zhang J, Gu D, Shi Z, Pan J, Geng Y, et al. Tirofiban facilitates the reperfusion process during endovascular thrombectomy in icas. *Exp Ther Med*. 2017;14:3314-3318
68. Lin L, Li W, Liu CC, Wu Y, Huang SH, Li XS, et al. Safety and preliminary efficacy of intravenous tirofiban in acute ischemic stroke patient without arterial occlusion on neurovascular imaging studies. *J Neurol Sci*. 2017;383:175-179
69. Li W, Lin L, Zhang M, Wu Y, Liu C, Li X, et al. Safety and preliminary efficacy of early tirofiban treatment after alteplase in acute ischemic stroke patients. *Stroke*. 2016;47:2649-2651

70. Zhu YQ, Zhang YJ, Ruan HL, Liu Q, Zhan Q, Li Q. Safety of tirofiban for patients with acute ischemic stroke in routine clinical practice. *Exp Ther Med*. 2015;10:169-174
71. Kellert L, Hametner C, Rohde S, Bendszus M, Hacke W, Ringleb P, et al. Endovascular stroke therapy: Tirofiban is associated with risk of fatal intracerebral hemorrhage and poor outcome. *Stroke*. 2013;44:1453-1455
72. Siebler M, Hennerici MG, Schneider D, von Reutern GM, Seitz RJ, Rother J, et al. Safety of tirofiban in acute ischemic stroke: The satis trial. *Stroke*. 2011;42:2388-2392
73. Torgano G, Zecca B, Monzani V, Maestroni A, Rossi P, Cazzaniga M, et al. Effect of intravenous tirofiban and aspirin in reducing short-term and long-term neurologic deficit in patients with ischemic stroke: A double-blind randomized trial. *Cerebrovasc Dis*. 2010;29:275-281
74. Seitz RJ, Hamzavi M, Junghans U, Ringleb PA, Schranz C, Siebler M. Thrombolysis with recombinant tissue plasminogen activator and tirofiban in stroke: Preliminary observations. *Stroke*. 2003;34:1932-1935
75. Guo Y, Lin Y, Tang Y, Tang Q, Wang X, Pan X, et al. Safety and efficacy of early antiplatelet therapy in acute ischemic stroke patients receiving endovascular treatment: A systematic review and meta-analysis. *J Clin Neurosci*. 2019;66:45-50
76. Zhou J, Gao Y, Ma QL. Safety and efficacy of tirofiban in acute ischemic stroke patients not receiving endovascular treatment: A systematic review and meta-analysis. *European review for medical and pharmacological sciences*. 2020;24(3):1492-1503.
77. Sun Y, Guo ZN, Yan X, Wang M, Zhang P, Qin H, et al. Safety and efficacy of tirofiban combined with endovascular therapy compared with endovascular therapy alone in acute ischemic stroke: A meta-analysis. *Neuroradiology*. 2021; 63(1):17-25
78. McClellan KJ, Goa KL. Tirofiban - a review of its use in acute coronary syndromes. *Drugs*. 1998;56:1067-1080

XVII. appendix

1. modified Rankin Scale (mRS)

The modified Rankin scale is used to measure the recovery of neurological function in patients after stroke. Boldface shows the formal definition of each level, and italics gives further guidance to reduce the possible errors between different observers, but there is no requirement for the architecture of the discussion. Please note that only symptoms that have occurred since stroke are considered. If the patient can walk with the help of some auxiliary devices without external help, it is considered to be able to walk independently.

If the two levels seem to be equally applicable to patients, and further questions are unlikely to make an absolutely correct choice, the more serious level should be selected.

0 No symptoms at all

Although there may be mild symptoms, the patient has not been aware of any new functional limitations and symptoms since stroke.

1 No significant disability despite symptoms, able to carry out all usual duties and activities

The patient has some symptoms caused by stroke, whether physical or cognitive (such as affecting speech, reading, writing; or physical movement; or feeling; or vision; or swallowing; or emotion), but can continue to engage in all work, social and leisure activities previously engaged in by stroke. The key question used to distinguish levels 1 and 2 (see below) can be, "is there something you used to do often, but you can't do it again after stroke?". Activities with a frequency of more than once a month are considered usual activities.

2 Slight disability, unable to carry out all previous activities but able to look after own affairs without assistance.

Some activities that can be completed before stroke (such as driving, dancing, reading or working) can no longer be performed by patients after stroke, but they can still take care of themselves every day without assistance from others. Patients can dress, walk, eat, go to the bathroom, prepare simple food, shop, travel locally, etc. without the help of others. Patients' lives need no supervision. Imagine that patients at this level can stay at home alone for a week or more without being cared for.

3 Moderate disability requiring some help, but able to walk without Assistance.

At this level, patients can walk independently (with the aid of walking aids), dress independently, go to the toilet, eat, etc., but more complex tasks need to be completed with the assistance of others. For example, the patient will need others to do shopping, cooking or cleaning instead, and visit patients more than once a week to ensure that the above activities are completed. What needs assistance is not only taking care of the body, but also giving advice. For example, patients at this level will need supervision or encouragement to deal with finance.

4 Moderate severe disability, unable to walk without assistance and unable to attend to own bodily needs without assistance.

Patients need other people to help with daily life, regardless of walking, dressing, going to the bathroom or eating. Patients need to be cared for at least once a day, usually twice or more, or must live close to the caregiver. To distinguish between levels 4 and 5 (see below), consider whether patients can routinely live alone for an appropriate time of the day.

5 Severe disability, bedridden incontinent, and requiring constant nursing care and attention.

Although there is no need for trained nurses, they need someone to look after them several times during the day and night.

6. death

2. National Institutes of Health Stroke Scale (NIHSS)

Assessment	Response	Score
**1a. Level of Consciousness:** The investigator must choose a response, even if a full evaluation is prevented by such obstacles as an endotracheal tube, language barrier, orotracheal trauma/bandages. A 3 is scored only if the patient makes no movement (other than reflexive posturing) in response to noxious stimulation.	0 = Alert; keenly responsive.<br>1 = Not alert, but arousable by minor stimulation to obey, answer, or respond.<br>2 = Not alert, requires repeated stimulation to attend, or is obtunded and requires strong or painful stimulation to make movements (not stereotyped).<br>3 = Responds only with reflex motor or autonomic effects or totally unresponsive, flaccid, areflexic.	
**1b. LOC Questions:** The patient is asked the month and his/her age. The answer must be correct - there is no partial credit for being close. Aphasic and stuporous patients who do not comprehend the questions will score 2. Patients unable to speak because of endotracheal intubation, orotracheal trauma, severe dysarthria from any cause, language barrier or any other problem not secondary to aphasia are given a 1. It is important that only the initial answer be graded and that the examiner not "help" the patient with verbal or non-verbal cues.	0 = Answers both questions correctly.<br>1 = Answers one question correctly.<br>2 = Answers neither question correctly.	
**1c. LOC Commands:** The patient is asked to open and close the eyes and then to grip and release the non-paretic hand. Substitute another one step command if the hands cannot be used. Credit is given if an unequivocal attempt is made but	0 = Performs both tasks correctly.<br>1 = Performs one task correctly.<br>2 = Performs neither task correctly.	

Assessment	Response	Score
not completed due to weakness. If the patient does not respond to command, the task should be demonstrated to them (pantomime) and score the result (i.e., follows none, one or two commands). Patients with trauma, amputation, or other physical impediments should be given suitable one-step commands. Only the first attempt is scored.		
**2. Best Gaze:** Only horizontal eye movements will be tested. Voluntary or reflexive (oculocephalic) eye movements will be scored but caloric testing is not done. If the patient has a conjugate deviation of the eyes that can be overcome by voluntary or reflexive activity, the score will be 1. If a patient has an isolated peripheral nerve paresis (CN III, IV or VI) score a 1. Gaze is testable in all aphasic patients. Patients with ocular trauma, bandages, pre-existing blindness or other disorder of visual acuity or fields should be tested with reflexive movements and a choice made by the investigator. Establishing eye contact and then moving about the patient from side to side will occasionally clarify the presence of a partial gaze palsy.	0 = Normal.<br>1 = Partial gaze palsy. This score is given when gaze is abnormal in one or both eyes, but where forced deviation or total gaze paresis are not present.<br>2 = Forced deviation, or total gaze paresis not overcome by the oculocephalic maneuver.	
**3. Visual:** Visual fields (upper and lower quadrants) are tested by confrontation, using finger	0 = No visual loss.<br>1 = Partial hemianopia.<br>2 = Complete hemianopia.<br>3 = Bilateral hemianopia (blind including	

Assessment	Response	Score
counting or visual threat as appropriate. Patient must be encouraged, but if they look at the side of the moving fingers appropriately, this can be scored as normal. If there is unilateral blindness or enucleation, visual fields in the remaining eye are scored. Score 1 only if a clear-cut asymmetry, including quadrantanopia is found. If patient is blind from any cause score 3. Double simultaneous stimulation is performed at this point. If there is extinction patient receives a 1 and the results are used to answer question 11.	cortical blindness).	
**4. Facial Palsy:**<br>Ask, or use pantomime to encourage the patient to show teeth or raise eyebrows and close eyes. Score symmetry of grimace in response to noxious stimuli in the poorly responsive or non-comprehending patient. If facial trauma/bandages, orotracheal tube, tape or other physical barrier obscures the face, these should be removed to the extent possible.	0 = Normal symmetrical movement.<br>1 = Minor paralysis (flattened nasolabial fold, asymmetry on smiling).<br>2 = Partial paralysis (total or near total paralysis of lower face).<br>3 = Complete paralysis of one or both sides (absence of facial movement in the upper and lower face).	

Assessment	Response	Score
**5 & 6. Motor Arm and Leg:**<br>The limb is placed in the appropriate position: extend the arms (palms down) 90 degrees (if sitting) or 45 degrees (if supine) and the leg 30 degrees (always tested supine). Drift is scored if the arm falls before 10 seconds or the leg before 5 seconds. The aphasic patient is encouraged using urgency in the voice and pantomime but not noxious stimulation. Each limb is tested in turn, beginning with the non-paretic arm. Only in the case of amputation or joint fusion at the shoulder or hip may the score be "9" and the examiner must clearly write the explanation for scoring as a "9".	0 = No drift, limb holds 90 (or 45) degrees for full 10 seconds.<br>1 = Drift, Limb holds 90 (or 45) degrees, but drifts down before full 10 seconds; does not hit bed or other support.<br>2 = Some effort against gravity, limb cannot get to or maintain (if cued) 90 (or 45) degrees, drifts down to bed, but has some effort against gravity.<br>3 = No effort against gravity, limb falls.<br>4 = No movement<br>9 = Amputation, joint fusion explain:	-
	**5a. Left Arm**	
	**5b. Right Arm**	
	0 = No drift, leg holds 30 degrees position for full 5 seconds.<br>1 = Drift, leg falls by the end of the 5 second period but does not hit bed.<br>2 = Some effort against gravity; leg falls to bed by 5 seconds, but has some effort against gravity.<br>3 = No effort against gravity, leg falls to bed immediately.<br>4 = No movement.<br>9 = Amputation, joint fusion explain:	-
	**6a. Left Leg**	
	**6b. Right Leg**	
**7. Limb Ataxia:**<br>This item is aimed at finding evidence of a unilateral cerebellar lesion. Test with eyes open. In case of visual defect, insure testing is done in intact visual field. The finger-nose-finger and heel-shin tests are performed on both sides, and ataxia is scored only if present out of proportion to weakness. Ataxia is absent in the patient who cannot understand or is paralyzed. Only	0 = Absent .<br>1 = Present in one limb .<br>2 = Present in two limbs If present, is ataxia in?	
	**Right arm** 1 = Yes 2 = No<br>9 = amputation or joint fusion, explain: ________________	-
	**Left arm** 1 = Yes 2 = No<br>9 = amputation or joint fusion, explain : ________________	-
	**Right leg** 1 = Yes 2 = No<br>9 = amputation or joint fusion, explain: ________________	-

Assessment	Response	Score
in the case of amputation or joint fusion may the item be scored "9", and the examiner must clearly write the explanation for not scoring. In case of blindness test by touching nose from extended arm position.	**Left leg** 1 = Yes 2 = No<br>9 = amputation or joint fusion, explain: ________________	-
**8. Sensory:**<br>Sensation or grimace to pin prick when tested, or withdrawal from noxious stimulus in the obtunded or aphasic patient. Only sensory loss attributed to stroke is scored as abnormal and the examiner should test as many body areas [arms (not hands), legs, trunk, face] as needed to accurately check for hemisensory loss. A score of 2, "severe or total," should only be given when a severe or total loss of sensation can be clearly demonstrated. Stuporous and aphasic patients will therefore probably score 1 or 0. The patient with brain stem stroke who has bilateral loss of sensation is scored 2. If the patient does not respond and is quadriplegic score 2. Patients in coma (item 1a=3) are arbitrarily given a 2 on this item.	0 = Normal; no sensory loss.<br>1 = Mild to moderate sensory loss; patient feels pinprick is less sharp or is dull on the affected side; or there is a loss of superficial pain with pinprick but patient is aware he/she is being touched.<br>2 = Severe to total sensory loss; patient is not aware of being touched in the face, arm, and leg.	
**9. Best Language:**<br>A great deal of information about comprehension will be obtained during the preceding sections of the examination. The patient is asked to describe what is happening in the attached picture, to name the items on the attached naming sheet, and to read from	0 = No aphasia, normal.<br>1 = Mild to moderate aphasia; some obvious loss of fluency or facility of comprehension, without significant limitation on ideas expressed or form of expression. Reduction of speech and/or comprehension, however, makes conversation about provided material difficult or impossible. For example in	

Assessment	Response	Score
the attached list of sentences. Comprehension is judged from responses here as well as to all of the commands in the preceding general neurological exam. If visual loss interferes with the tests, ask the patient to identify objects placed in the hand, repeat, and produce speech. The intubated patient should be asked to write. The patient in coma (question 1a=3) will arbitrarily score 3 on this item. The examiner must choose a score in the patient with stupor or limited cooperation but a score of 3 should be used only if the patient is mute and follows no one step commands.	conversation about provided materials examiner can identify picture or naming card from patient's response.<br>2 = Severe aphasia; all communication is through fragmentary expression; great need for inference, questioning, and guessing by the listener. Range of information that can be exchanged is limited; listener carries burden of communication. Examiner cannot identify materials provided from patient response.<br>3 = Mute, global aphasia; no usable speech or auditory comprehension.	
**10. Dysarthria:**<br>If patient is thought to be normal an adequate sample of speech must be obtained by asking patient to read or repeat words from the attached list. If the patient has severe aphasia, the clarity of articulation of spontaneous speech can be rated. Only if the patient is intubated or has other physical barrier to producing speech, may the item be scored "9", and the examiner must clearly write an explanation for not scoring. Do not tell the patient why he/she is being tested.	0 = Normal.<br>1 = Mild to moderate; patient slurs at least some words and, at worst, can be understood with some difficulty.<br>2 = Severe; patient's speech is so slurred as to be unintelligible in the absence of or out of proportion to any dysphasia, or is mute/anarthric.<br>9 = Intubated or other physical barrier, explain: ________________	
**11. Extinction and Inattention (formerly Neglect):**<br>Sufficient information to identify neglect may be obtained during	0 = No abnormality.<br>1 = Visual, tactile, auditory, spatial, or personal inattention or extinction to bilateral simultaneous stimulation in one	

Assessment	Response	Score
the prior testing. If the patient has a severe visual loss preventing visual double simultaneous stimulation, and the cutaneous stimuli are normal, the score is normal. If the patient has aphasia but does appear to attend to both sides, the score is normal. The presence of visual spatial neglect or anosagnosia may also be taken as evidence of abnormality. Since the abnormality is scored only if present, the item is never untestable.	of the sensory modalities.<br>2 = Profound hemi-inattention or hemi-inattention to more than one modality. Does not recognize own hand or orients to only one side of space.	
**TOTAL**		/
Additional item, not a part of the NIH Stroke Scale score.		-
**A. Distal Motor Function:** The patient's hand is held up at the forearm by the examiner and patient is asked to extend his/her fingers as much as possible. If the patient can't or doesn't extend the fingers the examiner places the fingers in full extension and observes for any flexion movement for 5 seconds. The patient's first attempts only are graded. Repetition of the instructions or of the testing is prohibited.	0 = Normal (No flexion after 5 seconds).<br>1 = At least some extension after 5 seconds, but not fully extended. Any movement of the fingers which is not command is not scored.<br>2 = No voluntary extension after 5 seconds. Movements of the fingers at another time are not scored.	-
	**a. Left Arm**	
	**b. Right Arm**	

Note: Please score according to the table and record the results. Do not change the score. The score reflects the actual situation of the patient, not what the doctor thinks the patient should be. Quick check and record the results. Don't train the patient (such as repeatedly asking the patient to make some effort) unless necessary guidance.

If some items are not evaluated, they shall be explained in detail in the table.

Attachment: drawings for Items 9 and 10 exam

Reading inspection Figure 1

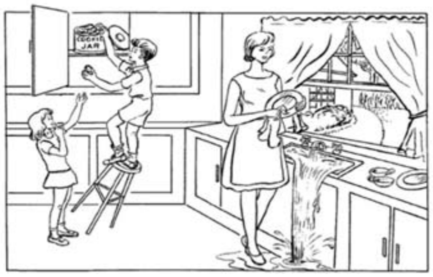

Reading inspection Fig. 2

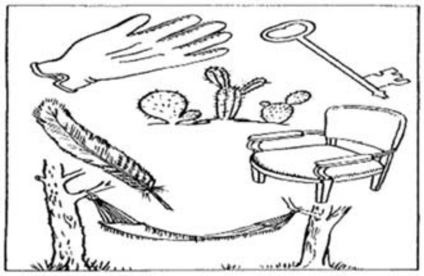

Reading inspection Figure 3

Please read the following sentences:

I know

Down the stairs

Go home and cook

Review at school

Deliver a wonderful speech

Reading inspection Figure 4

Please read out the following words:

Mom

earth

Aircraft aircraft

silk

Start work on time

Eat grapes without spitting their skins

Basic principles and precautions of NIHSS scoring

Basic principles of NIHSS scoring:

Record the patient's first response, even if the latter response may be better;

Note that only record what the patient does, not what you think they can do;

Record while checking, and try to avoid inducing patients;

For items that cannot be evaluated, please record the score as "9", which will be automatically processed according to the default value during computer statistical processing;

“Consistent” principle: pay attention to keeping the "consistent" evaluation standard during multiple follow-up visits.

How to evaluate the NIHSS score of comatose patients?

For patients with 1a score less than 3, each item should be evaluated one by one.

Only when the patient has no response to any harmful stimuli (rubbing the sternum,

pressing the orbit, etc.) with just reflex activity, item 1A will be rated as 3 points.

If 1a = 3 points, other items shall be rated as:

1b- awareness level question: 2 points

1c-consciousness level instruction: 2 points

2-gaze: according to whether it can be overcome by head eye reflex, if it can be overcome by head eye reflex, score 1 point; if not, score 2 points.

3- vision: assess with visual threat.

4- facial paralysis: 3 points

5. 6- limb movement: 4 points for each limb

7- ataxic exercise: the score can be given only when there is ataxia. If the patient's muscle strength decreases and he/she cannot complete the examination of finger pointing nose, heel, knee, tibia, etc., the score is 0.

8- sensation: 2 points

9- language: 3 points

10 dysarthria: 2 points

11- neglect: coma means losing all cognitive abilities, so 2 points are given.

How to calculate the total score of NIHSS?

In calculating the total score, the following items shall not be included in the total score:

Items 5 and 6 - "9 = amputation or joint fusion" in limb movement

Item 7 - the item to determine the location of ataxia in ataxia, that is, "left upper limb 1 = yes 2 = No 9 = amputation or joint fusion, explanation:" (not required for registration).

3. target artery recanalization grade (AOL grade):

Definition:
Grade 0
Total occlusion of targeted vessel
Grade 1
The targeted vessel is not completely occluded or partially recanalized, and there is no blood flow distally
Grade 2
The targeted vessel is not completely occluded or partially recanalized, and there is very little blood flow distally
Grade 3
The targeted vessel is completely recanalized and the distal flow is completely restored
