# Supplementary material for: Tirofiban on First-Pass Recanalization in Acute Stroke Endovascular Thrombectomy: The OPTIMISTIC Randomized Clinical Trial
Source: JAMA Netw Open. 2025 Apr 17;8(4):e255308. doi: 10.1001/jamanetworkopen.2025.5308 (PMC12006867; doi:10.1001/jamanetworkopen.2025.5308)
Supplement: Supplement 2. — eAppendix. List of Study Group and Trial Investigators eTable 1. List of Major Protocol Deviations in Intention-to-Treat Population eTable 2. Tirofiban Treatment Information in Intention-to-Treat Population eTable 3. Additional Procedural Characteristics of Intention-to-Treat Population eTable 4. Post Hoc Logistic Regression Analysis of Primary and Secondary Outcomes in Intention-to-Treat Population eTable 5. Outcomes of Per-Protocol Analysis eTable 6. Severe Adverse Events Summary of Intention-to-Treat Population eTable 7. Safety Outcomes of Safety Analysis Set eTable 8. Severe Adverse Events Summary of Safety Analysis Set eFigure 1. Study Timeline eFigure 2. Plot of First-Pass Thrombectomy Procedures Across Sites in Intention-to-Treat Population eFigure 3. Sensitivity Analysis With Missing Primary Outcome Data in Intention-to-Treat Population [file jamanetwopen-e255308-s002.pdf]

Supplemental Online Content

Lin L, Liu F, Yi T, et al. Tirofiban on first-pass recanalization in acute stroke endovascular thrombectomy: the OPTIMISTIC randomized clinical trial. *JAMA Netw Open*. 2025;8(4):e255308. doi:10.1001/jamanetworkopen.2025.5308

eAppendix. List of Study Group and Trial Investigators
eTable 1. List of Major Protocol Deviations in Intention-to-Treat Population
eTable 2. Tirofiban Treatment Information in Intention-to-Treat Population
eTable 3. Additional Procedural Characteristics of Intention-to-Treat Population
eTable 4. Post Hoc Logistic Regression Analysis of Primary and Secondary Outcomes in Intention-to-Treat Population
eTable 5. Outcomes of Per-Protocol Analysis
eTable 6. Severe Adverse Events Summary of Intention-to-Treat Population
eTable 7. Safety Outcomes of Safety Analysis Set
eTable 8. Severe Adverse Events Summary of Safety Analysis Set
eFigure 1. Study Timeline
eFigure 2. Plot of First-Pass Thrombectomy Procedures Across Sites in Intention-to-Treat Population
eFigure 3. Sensitivity Analysis With Missing Primary Outcome Data in Intention-to-Treat Population

This supplemental material has been provided by the authors to give readers additional information about their work.

© 2025 Lin L et al. *JAMA Network Open*.

eAppendix. List of Study Group and Trial Investigators

Trial Steering Committee
Prof Mark Parsons (Chair), Professor of Neurology at South Western Sydney Clinical Campuses, University of New South Wales, Sydney, NSW, Australia.

Prof Gang Li (Principal Investigator), Professor of Neurology at the Department of Neurology, Shanghai East Hospital, School of Medicine, Tongji University, Shanghai, China.

Prof Leonid Churilov, Professor of Biostatistics at Department of Medicine, University of Melbourne, Melbourne, VIC, Australia.

Data Safety Monitoring Board
Prof Christopher Levi (Chair), Professor of Neurology at John Hunter Health and Innovation Precinct Area Administration, New Lambton, NSW, Australia.

Prof Elizabeth Holiday, Professor of Biostatistics in the School of Medicine and Public Health at the University of Newcastle, Newcastle, NSW, Australia.

Prof Wusheng Zhu, Professor of Neurology at the Department of Neurology, Nanjing Jinling Hospital, Affiliated Hospital of Medical School, Nanjing University, Nanjing, China.

Statisticians
Dr Longting Lin, Senior Research Fellow at South Western Sydney Clinical Campuses, University of New South Wales, Sydney, NSW, Australia.

Prof Leonid Churilov, Professor of Biostatistics at Department of Medicine, University of Melbourne, Melbourne, Victoria, Australia.

Medical Review committee
Dr Feifeng Liu, and Dr Chen, Neurologists at the Department of Neurology, Shanghai East Hospital, School of Medicine, Tongji University, Shanghai, China

Imaging Analysis Group
Dr Yaping Xiao, Dr Hao Shen, Neurointerventionists at the Department of Neurology, Shanghai East Hospital, School of Medicine, Tongji University, Shanghai, China.

Dr Luran Xu, Neurologist at the Department of Neurology, Shanghai East Hospital, School of Medicine, Tongji University, Shanghai, China.

Dr Chushuang Chen, Research Fellow at South Western Sydney Clinical Campuses, University of New South Wales, Sydney, NSW, Australia.

Project management and imaging coordinating team
Dr Feifeng Liu, Neurologist at the Department of Neurology, Shanghai East Hospital, School of Medicine, Tongji University, Shanghai, China.

Contract research organization: Yi Ying Information Service (Quzhou) Technology Co. Ltd, Room 202-2, Building 4, 258 Huayuan Dong Road, Quzhou, Zhejiang, China.

Data Management and Programming
Contract research organization: Yi Ying Information Service (Quzhou) Technology Co. Ltd, Room 202-2, Building 4, 258 Huayuan Dong Road, Quzhou, Zhejiang, China.

© 2025 Lin L et al. *JAMA Network Open*.

Principal Investigators and Coordinators (center, with numbers of patients in parentheses)

Shanghai East Hospital (37): Gang Li, Yuming Long, Yue Zhang, Zhengyu Huang, Hao Zhang, Chenxin Jiang, Zhuojun Xu.

Shanghai Seventh People's Hospital (10): Feng Wang, Qiwei Wang, Yongpeng Wang, Min Yu, Wenhao Yang, Xiafei Wang, Wang Fu.

Zhangzhou Municipal Hospital (98): Wenhuo Chen, Tingyu Yi, Yanmin Wu, Dinglai Lin, Xiaohui Lin, Zhinan Pan, LiSan Zeng, Yuehong He, Xiaoyan Chen.

Jinan Central Hospital (14): Yanxin Zhao, Jialong Zhou, Fangzhuo Zhao, Tianrui Zhu, Dong Wang, Meilong Gao, Wanda Shi.

Shanghai Sixth People's Hospital (29): Yueqi Zhu, Jiangshan Deng, Haitao Lu, Liming Wei, Yi Gu, Yiran zhang.

Xuchang Central Hospital (5): Guangyu Xiang, Yali Liang, Zhe Qian, Ping Guo, Zhenzhen pan.

The First Affiliated Hospital of Ningbo University (7): Jianhong Yang, Yuefei Wu, Yueshi Huang, Renshuai Liu, Chao Wei, Dong Han.

© 2025 Lin L et al. *JAMA Network Open*.

eTable 1. List of Major Protocol Deviations in Intention-to-Treat Population

NO.	Patient ID	group	Summary	Description of protocol deviations
1	TF010013	Tirofiban	Inaccurate dose of study drug	The dose of tirofiban (0.1 mg intravenous bolus) was lower than the recommended dose in the protocol (0.48 mg intravenous bolus)
2	TF030003	Control	Violating exclusion criteria	Atrial fibrillation was diagnosed with the delayed report of electrocardiogram
3	TF040002	Control	Missing key imaging data	DSA image was not available
4	TF060018	Control	Violating exclusion criteria	Atrial fibrillation was diagnosed with the delayed report of electrocardiogram
5	TF040003	Tirofiban	Thrombectomy procedure not performed	The subject was unsuitable for thrombectomy procedure.
6	TF070002	Tirofiban	Thrombectomy procedure not performed	The subject was unsuitable for thrombectomy procedure.
7	TF070005	Control	Thrombectomy procedure not performed	The subject was unsuitable for thrombectomy procedure.
8	TF060059	Tirofiban	Violating exclusion criteria	Atrial fibrillation was diagnosed with the delayed report of electrocardiogram
9	TF090004	Tirofiban	Study drug not delivered	Intravenous tirofiban was not used as assigned
10	TF090005	Tirofiban	Violating exclusion criteria	The subject had a medical history of atrial fibrillation
11	TF090005	Tirofiban	Study drug not delivered	Intravenous tirofiban was not used as assigned
12	TF090006	Tirofiban	Violating inclusion criteria	The subject was 88 years old and did not meet the inclusion criteria of age (18-85 years old)
13	TF040018	Control	Thrombectomy procedure not performed	The subject was unsuitable for thrombectomy procedure.
14	TF090006	Tirofiban	Thrombectomy procedure not performed	The subject was unsuitable for thrombectomy procedure.

© 2025 Lin L et al. *JAMA Network Open*.

eTable 2. Tirofiban Treatment Information in Intention-to-Treat Population

	**Tirofiban group (n=102)**
**Protocol recommendation: Intravenous bolus of Tirofiban at the dose of 10 μg/kg**	
No tirofiban delivery, n (%)	2 (2)
Tirofiban bolus dose < 10 μg/kg, n (%)	1 (1)
**Protocol recommendation: Intravenous infusion of Tirofiban at the dose of 0.1μg/kg/min for 24 hours**	
No tirofiban delivery, n (%)	2 (2)
Tirofiban infusion stop early, n (%)	9 (9)
**Reasons for early stop of intravenous tirofiban infusion**	
Hemorrhagic transformation, n (%)	4 (4)
Contrast extravasation, n (%)	3 (3)
Suspected cerebral embolism, n (%)	1 (1)
Pancytopenia, n (%)	1 (1)

© 2025 Lin L et al. *JAMA Network Open*.

eTable 3. Additional Procedural Characteristics of Intention-to-Treat Population

	Control group (n=98)	Tirofiban group (n=102)
**Residual stenosis after thrombectomy, n/N (%)[a]**		
≥ 50%	69/93 (74)	66/97 (68)
< 50%	24/93 (26)	31/97 (32)
**Time from groin puncture to procedure completion (minutes), median (IQR)**	105.5 (75-153)	96.5 (69-133)
**EVT procedure complications, n/N (%)[b]**		
Clot migration	10/97 (10)	10/102 (10)
Arterial dissection	1/97 (1)	2/102 (2)
Contrast media extravasation	7/97 (7)	4/102 (4)
**mTICI grade after procedure completion, n/N (%)[b]**		
0	2/97 (2)	3/102 (3)
1	2/97 (2)	2/102 (2)
2a	4/97 (4)	1/102 (1)
2b	24/97 (25)	24/102 (24)
3	65/97 (67)	72/102 (71)

mTICI refers to modified Treatment in Cerebral Ischaemia scale, with higher grade indicating greater reperfusion.
a. Data were not available for 10 patients; thrombectomy was not performed on 5 patients (3 in the tirofiban group and 2 in the control group), DSA data was not available for 1 patient in the control group, and 4 patients had no residual stenosis information (2 in tirofiban and 2 in control).
b. DSA data was not available for 1 patient in the control group.

eTable 4. Post Hoc Logistic Regression Analysis of Primary and Secondary Outcomes in Intention-to-Treat Population

	**Control group (N=98)**	**Tirofiban group (N=102)**	**Effect size [95% CI]**	**P value**
**Primary outcome**				
First-pass recanalization without symptomatic intracranial hemorrhage, n/N (%)[a]	46/95 (48)	64/99 (65)	Odds Ratio[e]: 1.96 [1.10-3.50]	0.02
**Secondary efficacy outcomes**				
First-pass recanalization, n/N (%)[a]	48/95 (51)	64/99 (65)	Odds Ratio[e]: 1.82 [1.02-3.26]	..
Recanalization after thrombectomy, n/N (%)[a]	76/95 (80)	89/99(90)	Odds Ratio[e]: 1.83 [0.85-3.92]	..
Recanalization at the end of endovascular procedure, n/N (%)[b]	89/97 (92)	96/102 (94)	Odds Ratio[f]: 1.50 [0.48-4.74]	..
Recanalization at 24-72 hours, n/N (%)[c]	62/69 (90)	65/70 (93)	Odds Ratio[f]: 1.66 [0.47-5.84]	..
90-day mRS 0-2, n/N (%)[d]	60/98 (61)	53/101 (53)	Odds Ratio[g]: 0.62 [0.35-1.19]	..
**Safety outcomes**				
Mortality at 90 days, n/N (%)[d]	11/98 (11)	13/101 (13)	Odds Ratio[g]: 1.22 [0.50-2.95]	..

a. Data were not available for 6 patients; thrombectomy was not performed on 5 patients (3 in the tirofiban group and 2 in the control group), and DSA data was not available for 1 patient in the control group.
b. DSA data was not available for 1 patient in the control group.
c. Data for follow-up CTA or MRA were not available for 61 patients (32 in the tirofiban group and 29 in the control group).
d. One patient was lost to follow-up at 90 days in the tirofiban group.
e. treatment effect was adjusted for time from onset or last known well to randomization and occlusion site.
f. treatment effect was adjusted for time from onset or last known well to randomization, occlusion site, and rescue treatment.
g. treatment effect was adjusted for age, time from onset or last known well to randomization, and baseline NIHSS.

© 2025 Lin L et al. *JAMA Network Open*.

eTable 5. Outcomes of Per-Protocol Analysis

	**Control group (N=93)[a]**	**Tirofiban group (N=95)[a]**	**Effect size [95% CI]**	**P value**
**Primary outcome**				
First-pass recanalization without symptomatic intracranial haemorrhage, n/N (%)	45/93 (48)	64/95 (67)	Adjusted Risk Ratio[d]: 1.40 [1.08-1.80]	0.01
**Secondary efficacy outcomes**				
First-pass recanalization, n/N (%)	47/93 (51)	64/95 (67)	Adjusted Risk Ratio[d]: 1.34 [1.05-1.72]	..
Recanalization after thrombectomy, n/N (%)	75/93 (81)	86/95 (91)	Adjusted Risk Ratio[d]: 1.12 [1.00-1.27]	..
Number of thrombectomy passes, median (IQR)	1 (1-2)	1 (1-2)	Adjusted Coefficient[d]: 0 [-0.21-0.21]	..
Recanalization after all endovascular procedure (stenting/angioplasty), n/N (%)	87/93 (94)	90/95 (95)	Adjusted Risk Ratio[e]: 1.01 [0.95-1.08]	..
Recanalization at 24-72 hours, n/N (%)[b]	60/67 (90)	63/68 (93)	Adjusted Risk Ratio[e]: 1.04 [0.94-1.14]	..
90-day mRS 0-2; n/N (%)[c]	58/93 (62)	50/94 (53)	Adjusted Risk Ratio[f]: 0.85 [0.67-1.07]	..
90-day ordinal mRS, median (IQR)[c]	2 (1-4)	2 (1-4)	Adjusted Common Odds Ratio[f]: 0.69 [0.42-1.17]	..
**Safety outcomes**				
Symptomatic intracranial haemorrhage, n/N (%)	5/93 (5)	0/95 (0)	Unadjusted Risk Difference[g]: -0.05 [-0.10 - -0.01]	..
Mortality at 90 days, n/N (%)	10/93 (11)	10/94 (11)	Adjusted Risk Ratio[f]: 1.04 [0.46-2.37]	..

a. 12 patients were excluded from per protocol analysis for major protocol deviations (5 in the control group and 7 in the tirofiban group).
b. Data for follow-up CTA or MRA were not available for 53 patients (26 in the control group and 27 in the tirofiban group).
c. One patient was lost to follow-up at 90 days in the tirofiban group.
d. treatment effect was adjusted for time from onset or last known well to randomisation and occlusion site.
e. treatment effect was adjusted for time from onset or last known well to randomisation, occlusion site, and rescue treatment.
f. treatment effect was adjusted for age, time from onset or last known well to randomisation, and baseline NIHSS.
g. Unadjusted risk difference was reported; relative risk was not measurable since the sICH rate was 0 in the tirofiban group.

© 2025 Lin L et al. *JAMA Network Open*.

eTable 6. Severe Adverse Events Summary of Intention-to-Treat Population

	Control group (n=19)	Tirofiban group (n=16)
**SAE categories, n/N (%)**		
Cause death	11/19 (58)	14/16 (88)
Need hospitalization or extension of hospitalization	3/19 (16)	1/16 (6)
Major medical event	1/19 (5)	1/16 (6)
Life threatening event	4/19 (21)	0/16 (0)
**Relationship of SAE to the investigational drug, n/N (%)**		
Not related to	17/19 (89)	13/16 (81)
May not be related	1/19 (5)	1/16 (6)
May be related to	1/19 (5)	2/16 (13)

© 2025 Lin L et al. *JAMA Network Open*.

eTable 7. Safety Outcomes of Safety Analysis Set

	**Control group (N=100)[a]**	**Tirofiban group (N=100)[a]**	**Effect size [95% CI]**
symptomatic intracranial haemorrhage, n/N (%)[b]	6/99 (6)	0/100 (0)	Unadjusted Risk Difference[d]: -0.06 [-0.11 to -0.01]
Mortality at 90 days, n/N (%)[c]	12/100 (12)	12/99 (12)	Adjusted Relative Risk[e]: 1.08 [0.52 to 2.24]

a. two patients were randomised to the tirofiban group but did not receive the assigned treatment; they were crossed over to the control group for safety analysis.
b. Data for follow-up NCCT was not available for 1 patient in the control group.
c. One patient was lost to follow-up at 90 days in the tirofiban group.
d. Unadjusted risk difference was reported; relative risk was not measurable since the sICH rate was 0 in the tirofiban group.
e. treatment effect was adjusted for age, time from onset or last known well to randomisation, and baseline NIHSS.

© 2025 Lin L et al. *JAMA Network Open*.

eTable 8. Severe Adverse Events Summary of Safety Analysis Set

	**Control group (n=20)**	**Tirofiban group (n=15)**
**SAE categories, n/N (%)**		
Cause death	12/20 (60)	13/15 (87)
Need hospitalization or extension of hospitalization	3/20 (15)	1/15 (7)
Major medical event	1/20 (5)	1/15 (7)
Life threatening event	4/20 (20)	0/15 (0)
**Relationship of SAE to the investigational drug, n/N (%)**		
Not related to	18/20 (90)	12/15 (80)
May not be related	1/20 (5)	1/15 (7)
May be related to	1/20 (5)	2/15 (13)

© 2025 Lin L et al. *JAMA Network Open*.

eFigure 1. Study Timeline

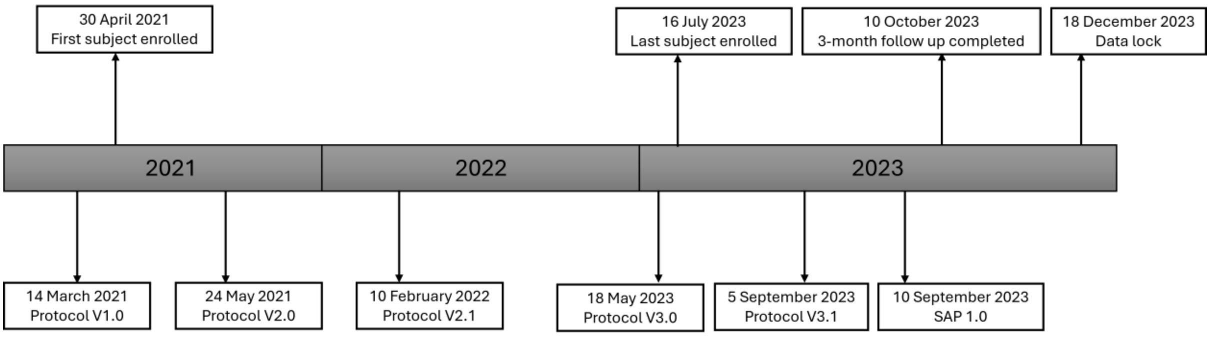

© 2025 Lin L et al. *JAMA Network Open*.

eFigure 2. Plot of First-Pass Thrombectomy Procedures Across Sites in Intention-to-Treat Population

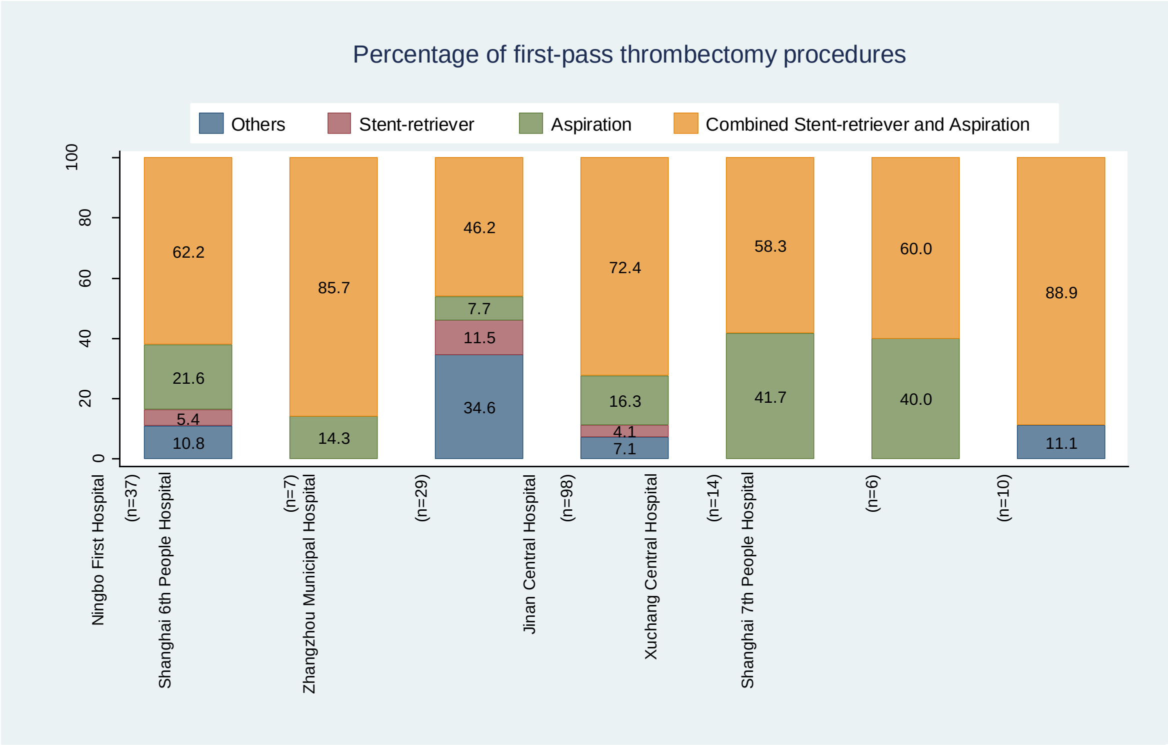

© 2025 Lin L et al. *JAMA Network Open*.

eFigure 3. Sensitivity Analysis With Missing Primary Outcome Data in Intention-to-Treat Population

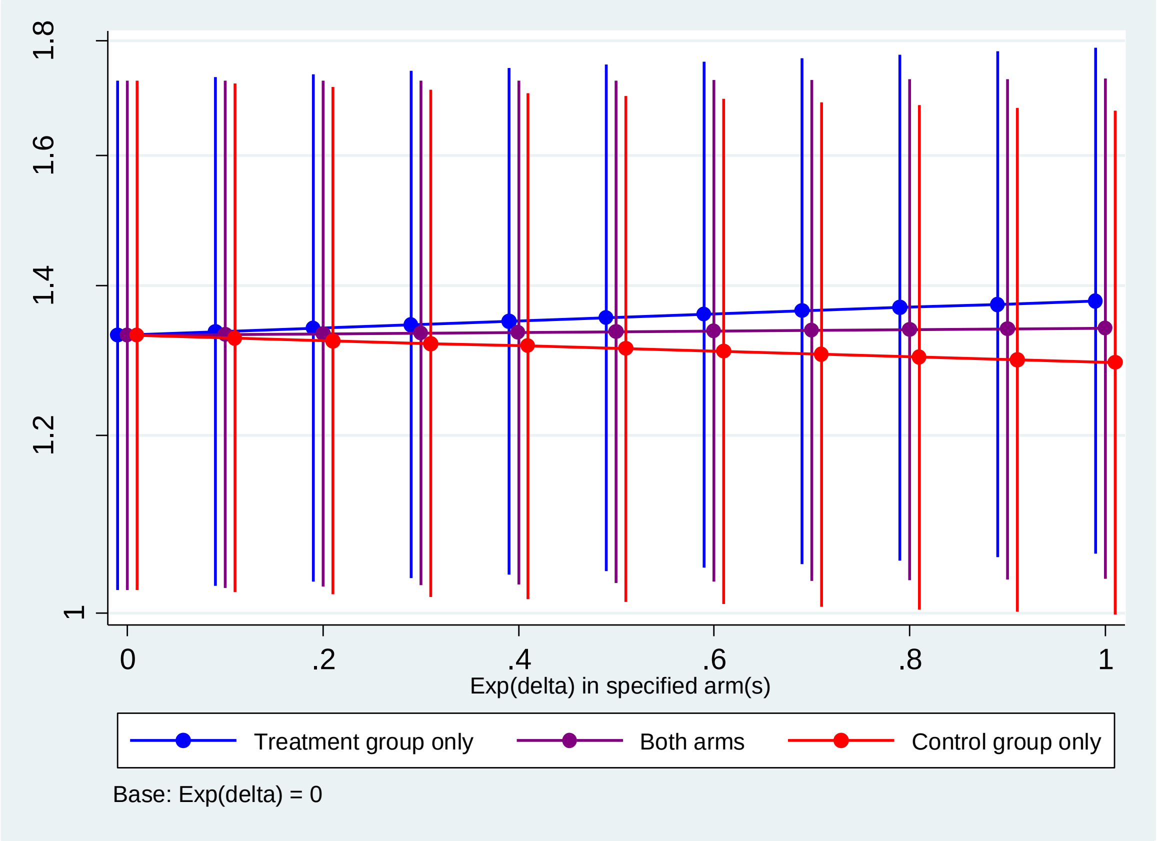

The positive effect of intervention remains statistically significant (lower end of 95% confidence interval above 1) across the full range of tested assumptions regarding missingness at random.

© 2025 Lin L et al. *JAMA Network Open*.
