# Supplementary material for: Tirofiban on First-Pass Recanalization in Acute Stroke Endovascular Thrombectomy: The OPTIMISTIC Randomized Clinical Trial
Source: JAMA Netw Open. 2025 Apr 17;8(4):e255308. doi: 10.1001/jamanetworkopen.2025.5308 (PMC12006867; doi:10.1001/jamanetworkopen.2025.5308)
Supplement: Supplement 3. — Nonauthor Collaborators [file jamanetwopen-e255308-s003.pdf]

Supplemental Online Content: Nonauthor Collaborators
*First name, last name, and suffix (if applicable) are required and will appear in PubMed.

*Group Name(s): The OPTIMISTIC Investigators							
***First Name and Middle Initial(s)**	***Last Name**	***Suffix (eg, Jr, III)**	**Academic Degrees**	**Institution**	**Location (city, state/province, country)**	**Role or Contribution, eg, chair, principal investigator**	**Group (if more than 1 Group listed in the byline) and/or Subgroup (eg, Steering Committee)**
Yuming	Long		MD	Shanghai East Hospital	Shanghai, China	Patient recruitment	NA
Yue	Zhang		MD	Shanghai East Hospital	Shanghai, China	clinical data collection	NA
Zhengyu	Huang		MD	Shanghai East Hospital	Shanghai, China	clinical data collection	NA
Hao	Zhang		MM	Shanghai East Hospital	Shanghai, China	patient follow-UP	NA
Chenxin	Jiang		MM candidate	Shanghai East Hospital	Shanghai, China	Imaging data collection	NA
Zhuojun	Xu		B.S.N.	Shanghai East Hospital	Shanghai, China	Study drugs management	NA
Qiwei	Wang		MM	Shanghai Seventh People's Hospital	Shanghai, China	Patient recruitment	NA
Yongpeng	Wang		MM	Shanghai Seventh People's Hospital	Shanghai, China	Patient recruitment	NA
Min	Yu		MM	Shanghai Seventh People's Hospital	Shanghai, China	clinical data collection	NA
Wenhao	Yang		MM	Shanghai Seventh People's Hospital	Shanghai, China	clinical data collection	NA
Xiafei	Wang		MM	Shanghai Seventh People's Hospital	Shanghai, China	Imaging data collection	NA
Wang	Fu		MM	Shanghai Seventh People's Hospital	Shanghai, China	Imaging data collection	NA
Yanmin	Wu		MB	Zhangzhou Municipal Hospital	Fujian, China	Patient recruitment	NA
Dinglai	Lin		MB	Zhangzhou Municipal Hospital	Fujian, China	Patient recruitment	NA
Xiaohui	Lin		MM	Zhangzhou Municipal Hospital	Fujian, China	clinical data collection	NA
Zhinan	Pan		MB	Zhangzhou Municipal Hospital	Fujian, China	clinical data collection	NA
Lisan	Zeng		MM	Zhangzhou Municipal Hospital	Fujian, China	Imaging data collection	NA
Yuehong	He		B.S.N.	Zhangzhou Municipal Hospital	Fujian, China	Study drugs management	NA
Xiaoyan	Chen		B.S.N.	Zhangzhou Municipal Hospital	Fujian, China	Study drugs management	NA
Jialong	Zhou		MM	Jinan Central Hospital	Shandong, China	Patient recruitment	NA
Fangzhuo	Zhao		MM	Jinan Central Hospital	Shandong, China	Patient recruitment	NA
Tianrui	Zhu		MD	Jinan Central Hospital	Shandong, China	clinical data collection	NA
Dong	Wang		MM	Jinan Central Hospital	Shandong, China	clinical data collection	NA
Meilong	Gao		MM	Jinan Central Hospital	Shandong, China	Imaging data collection	NA

Supplemental Online Content: Nonauthor Collaborators
*First name, last name, and suffix (if applicable) are required and will appear in PubMed.

*First Name and Middle Initial(s)	*Last Name	*Suffix (eg, Jr, III)	Academic Degrees	Institution	Location (city, state/province, country)	Role or Contribution, eg, chair, principal investigator	Group (if more than 1 Group listed in the byline) and/or Subgroup (eg, Steering Committee)
Wanda	Shi		MD	Jinan Central Hospital	Shandong, China	Imaging data collection	NA
Jiangshan	Deng		MD	Shanghai Sixth People's Hospital	Shanghai, China	Patient recruitment	NA
Haitao	Lu		MD	Shanghai Sixth People's Hospital	Shanghai, China	Patient recruitment	NA
Liming	Wei		MD	Shanghai Sixth People's Hospital	Shanghai, China	Patient recruitment	NA
Yi	Gu		MM	Shanghai Sixth People's Hospital	Shanghai, China	clinical data collection	NA
Yiran	Zhang		MD	Shanghai Sixth People's Hospital	Shanghai, China	Imaging data collection	NA
Zhigang	He		MM	Xuchang Central Hospital	Henan, China	Patient recruitment	
Yali	Liang		MM	Xuchang Central Hospital	Henan, China	Patient recruitment	NA
Zhe	Qian		MM	Xuchang Central Hospital	Henan, China	clinical data collection	NA
Ping	Guo		MB	Xuchang Central Hospital	Henan, China	Imaging data collection	NA
Zhenzhen	Pan		B.S.N.	Xuchang Central Hospital	Henan, China	Study drugs management	NA
Yuefei	Wu		MM	The first Affiliated Hospital of Ningbo University	Zhejiang, China	Patient recruitment	NA
Yueshi	Huang		MS	The first Affiliated Hospital of Ningbo University	Zhejiang, China	Patient recruitment	NA
Renshuai	Liu		MM	The first Affiliated Hospital of Ningbo University	Zhejiang, China	clinical data collection	NA
Chao	Wei		MM	The first Affiliated Hospital of Ningbo University	Zhejiang, China	clinical data collection	NA
Dong	Han		MM	The first Affiliated Hospital of Ningbo University	Zhejiang, China	Imaging data collection	NA
