# Supplementary material for: Tirofiban on First-Pass Recanalization in Acute Stroke Endovascular Thrombectomy: The OPTIMISTIC Randomized Clinical Trial
Source: JAMA Netw Open. 2025 Apr 17;8(4):e255308. doi: 10.1001/jamanetworkopen.2025.5308 (PMC12006867; doi:10.1001/jamanetworkopen.2025.5308)
Supplement: Supplement 4. — Data Sharing Statement [file jamanetwopen-e255308-s004.pdf]

Data Sharing Statement

Lin. Tirofiban on First-Pass Recanalization in Acute Stroke Endovascular Thrombectomy. *JAMA Netw Open*. Published April 17, 2025. doi:10.1001/jamanetworkopen.2025.5308

Data
Additional Information: ClinicalTrials.gov Identifier: NCT04851457
Data available: Yes
Data types: Deidentified participant data
How to access data: Data access can be requested by contacting the corresponding author Dr Gang Li, email address: ligang@tongji.edu.cn.
When available: With publication

Supporting Documents
Document types: Statistical/analytic code
How to access documents: Access to statistical code can be requested by contacting the corresponding author Dr Gang Li, email address: ligang@tongji.edu.cn.
When available: With publication

Additional Information
Who can access the data: Researchers whose proposed use of the data has been approved.
Types of analyses: For research purpose.
Mechanisms of data availability: Access to trial data will be made available after approval of a proposal by the trial steering committee with a signed data access agreement.
